# metaFun: An analysis pipeline for metagenomic big data with fast and unified functional searches

**DOI:** 10.1080/19490976.2025.2611544

**Published:** 2026-01-13

**Authors:** Hyeon Gwon Lee, Ju Yeon Song, Jaekyung Yoon, Yusook Chung, Soon-Kyeong Kwon, Jihyun F. Kim

**Affiliations:** a Department of Systems Biology, Division of Life Sciences, and Institute for Life Science and Biotechnology, Yonsei University, Seoul, Republic of Korea; b R&D Center, Cell Biotech Co., Ltd., Gimpo, Republic of Korea; c Division of Applied Life Science (BK21), Gyeongsang National University, Jinju, Republic of Korea; d Microbiome Initiative, Yonsei University, Seoul, Republic of Korea

**Keywords:** Whole metagenome sequence, taxonomic classification, interactive visualization, standard operating procedure, reproducibility

## Abstract

Metagenomic approaches offer unprecedented opportunities to characterize microbial community structure and function, yet several challenges remain unresolved. Inconsistent genome quality impairs reliability of metagenome-assembled genomes, lack of unified taxonomic criteria limits cross-study comparability, and multi-step workflows involving numerous programs and parameters hinder reproducibility and accessibility. We benchmarked existing programs and parameters using simulated metagenomic data to identify optimal configurations. metaFun is an open-source, end-to-end pipeline that integrates quality control, taxonomic profiling, functional profiling, *de novo* assembly, binning, genome assessment, comparative genomic analysis, pangenome annotation, network analysis, and strain-level microdiversity analysis into a unified framework. Interactive modules support standardized data interpretation and exploratory visualization. The pipeline is implemented with Nextflow and containerized with Apptainer, ensuring environment reproducibility and scalability. Comprehensive documentation is available at https://metafun-doc.readthedocs.io/en/main. The pipeline was validated using a colorectal cancer cohort dataset. By addressing key methodological gaps, metaFun facilitates accessible and reproducible metagenomic analysis for the broader research community.

## Graphical abstract

**Figure:**
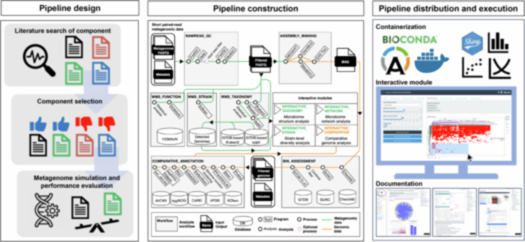

## Introduction

Metagenome analysis has emerged as a crucial technique to understand microbes in diverse environments. Metagenome-assembled genomes (MAGs) drawn from whole metagenome sequencing led to the creation of extensive MAG catalogs^1^^,^^2^ expanding knowledge of microbial functional diversity, genomic evolution, and their physiological and ecological roles. While MAGs help retrieve some uncultivated microbes for experimental studies,^3^^,^^4^ a substantial proportion of microbial genomes remains untapped as microbial dark matter.^5^ The landscape of computational tools for metagenomic analysis continues to evolve to uncover this information. However, a single factor can significantly alter analysis results. For instance, varying confidence thresholds of Kraken2 can alter taxonomic profiling accuracy by up to 50% in both precision and recall.^6^ Program versions, parameters, and operation systems also affect result consistency in metagenomic analysis.^7^^,^^8^ Furthermore, careful interpretation of MAGs is vital. A significant variation in core and accessory genes was observed even using high-quality MAGs within a species.^9^ Chimeric sequences incorporated from other strains or species are a particular concern.^10^ These discrepancies can have profound implications for downstream analyses and may lead to misinterpretations of functional capabilities and ecological roles.^11^^,^^12^ Robust metagenomic data analysis requires consideration of key factors such as database and reference genome selection, taxonomic and functional assignment, and efficiency of large-scale data analysis.^13^

These challenges have spurred initiatives like Critical Assessment of Metagenome Interpretation (CAMI) II^14^ to characterize best practices by extensive benchmarking. Numerous methods have been proposed for standardized analysis,^15^^,^^16^ but consistent analysis requires controlling software versions, parameters, and computing environments. Several methodologies have been developed to ensure reproducible and consistent analysis.^17^ Workflow managers, package managers, and containers successfully facilitate the construction of analysis pipelines that abide by FAIR (Findability, Accessibility, Interoperability, and Reusability) principles.^18^ Some pipelines are optimized for metagenomic sequence and MAG generation.^19‐24^ These tools encompass multiple steps, including quality control, assembly, binning, MAG quality assessment, and taxonomic and functional annotation of metagenomic reads and MAGs. However, existing pipelines often requirer bioinformatics skills, manual analysis environment setup and resource-intensive computation. Although interactive analytical programs with graphical user interfaces enhance researchers’ data interpretation,^25^^,^^26^ their visualization capabilities are often disconnected from data generation processes, impeding the unified execution of data generation and exploratory analysis.

We introduce metaFun, a whole metagenomic data analysis pipeline emphasizing scalability and accuracy with an optimized standard operating procedure. To comply with FAIR principles, the pipeline was built using Apptainer (formerly Singularity) and Conda, ensuring seamless operation through an open-source framework. Moreover, interactive visualization modules enable faster data comprehension and exploratory data analysis. By combining robust analysis methods with user-friendly interfaces, metaFun supports researchers to easily and efficiently process metagenomic data, generate high-quality MAGs, and perform sophisticated comparative analyses with reproducibility and scalability.

## Materials and methods

### Program and database selection criteria

We selected programs and databases based on several criteria represented in Supplementary Note 1 A. All components of metaFun modules were established based on literature reviews and the performances of some components were evaluated in this study (Supplementary Note 1B-1I). Programs and databases used for pipeline construction are listed in Table S1.

### Simulation of metagenomic reads using CAMI profiles

Fifteen microbial taxonomic abundance profiles with five samples from each of the three environments (human gut, rhizosphere, and marine) from the CAMI challenge^14^ were retrieved. The conversion process from NCBI to Genome Taxonomy Database (GTDB) taxonomy^27^ and metagenome simulation process are detailed in Supplementary Note 2 A.

### Performance evaluation of *de novo* assembly and binning/refining methods

Two *de novo* assemblers, MEGAHIT^28^ and MetaSPAdes^29^ were utilized for performance evaluation of *de novo* assembly using MetaQUAST.^30^ For the binning process, MetaBAT2 v2.15,^31^ SemiBin2 v2.1.0,^32^ and DAS Tool v1.1.7^33^ were evaluated for their performance using AMBER.^34^

To generate genomes that emulate MAG features, 3,350 genomes were randomly fragmented to make a “Fragmented” genome dataset, and further removal of 5% from each contig end resulted in a “Fragmented Incomplete” dataset. We refer to these datasets as “Complete”, “Fragmented”, and “Fragmented Incomplete” in the main text. Detailed methods are available in Supplementary Note 2B. MAG quality was classified based on minimum information about a metagenome-assembled genome (MIMAG) standards.^35^ High-quality MAGs were defined as those with ≥90% completeness and <5% contamination, while medium-quality MAGs required ≥50% completeness and <10% contamination, as assessed by CheckM2. The additional MIMAG criteria for rRNA and tRNA gene presence were not applied, as these genes are often poorly recovered in short-read metagenomic assemblies due to their repetitive sequences.

### Evaluation of the impact of parameter selection on pangenome-based functional annotation and comparative genomic analysis

Coding sequences (CDSs) were predicted using Prokka v1.14.6,^36^ with PPanGGOLiN v2.0.5^37^ for comparative genomic analysis. Gene families were clustered using two thresholds (80% identity 80% coverage and 90% identity 50% coverage), and functionally annotated using KOfamScan v1.3.0^38^ with the KOfam database. Annotation accuracy was evaluated using normalized Manhattan distance and F1 score metrics. Detailed metrics and methods are available in Supplementary Note 2 C.

### Evaluation of the impact of parameter selection on genome fluidity and core genome analysis

Genome fluidity was calculated using PPanGGOLiN, with ten species selected to evaluate the accuracy of core genome tree inference across varying gene family clustering thresholds and genome qualities. Phylogenetic trees were constructed using concatenated core gene alignments (90%, 95%, 99% thresholds) with FastTree v2.1.11.^39^ Tree topologies were compared to assess the impact of different parameters on inference accuracy through patristic distance matrices and Mantel tests. Detailed methods are available in Supplementary Note 2D.

### Construction of taxonomy-profiling databases with GTDB r220 and detected genomes, and performance evaluation of varying parameters

For taxonomic profiling of metagenomes, databases were constructed using 113,104 representative genomes from GTDB r220 for Kraken2 v2.1.3,^40^ Bracken v2.9,^41^ and sylph v0.6.1.^42^ Gold taxonomic and sequence abundance profiles were generated with CAMISIM^43^ and abundance calculation. Profiling accuracy was evaluated by adjusting Kraken2 confidence values, Bracken relative abundance thresholds, and sylph parameters, and assessing L1 norm, Bray-Curtis dissimilarity, and F1 score with OPAL v1.0.12.^44^ Detailed methods are available in Supplementary Note 2E.

### Analysis with a real metagenomic dataset

For a case study, a raw metagenomic dataset from healthy controls (CTRL) and colorectal cancer (CRC) patients under accession PRJNA447983^45^ was downloaded using sra-tools 3.0.10. Metagenomic data from CTRL and CRC was utilized for analysis (*n* = 113, Table S15). The metagenomic dataset was trimmed, quality-controlled, and host-decontaminated using WMS_TAXONOMY, and then taxonomic compositions were profiled using sylph with GTDB r220 database. Alpha and beta diversity and differential abundance analysis were performed using the interactive module of WMS_TAXONOMY. For CRC-associated species identification, a linear mixed effect model was applied using MaAsLin2, with disease, age, and sex as fixed effects. We applied 10% prevalence threshold without relative abundance filtration and log10 transformation. *P* values were corrected using Benjamini-Hochberg method. From 6,598 MAGs generated, 2,776 genomes meeting medium-quality criteria were inspected using GENOME SELECTOR. We selected *Bacteroides uniformis* to show demonstration of the interactive module that utilizes COMPARATIVE_ANNOTATION. We applied Fisher's exact test to identify the associations between genes and health status metadata of *B. uniformis* MAGs.

To assess population microdiversity of the dataset, taxonomic profile from the WMS_TAXONOMY module was used as input for the WMS_STRAIN module. Reads were aligned to these references using Bowtie2 with default parameters. Population microdiversity profiling was performed with inStrain,^46^ applying filters for coverage at least 5 and breadth at least 50% to ensure reliable variant calling. Key metrics included single nucleotide variant (SNV) density, nucleotide diversity, and genome-wide/gene-level ratio of nonsynonymous to synonymous polymorphisms (pN/pS). Strain sharing across samples was identified via population-level average nucleotide identity (popANI) at a 99.999% threshold. Functional annotations from eggNOG were integrated to analyze COG category distributions at specific taxonomic rank, assessing disease-associated shifts in evolutionary dynamics.

For network analysis, taxonomic profiles with metadata were analyzed using the INTERACTIVE_NETWORK module. Microbial co-occurrence networks were constructed using FlashWeave^47^ on genus-level aggregated abundances. Global topology metrics (node/edge counts, density, average degree, clustering coefficient, modularity) were computed via igraph,^48^ with modularity derived from the fast greedy clustering algorithm. Each node's role and influence within the network was evaluated using participation coefficient (Pi), within-module degree (Zi),^49^ and Integrated Value of Influence (IVI).^50^ Network robustness was evaluated with brainGraph^51^ through 100 averaged random removal iterations and targeted attacks involving sequential removal by descending degree, betweenness, or IVI, quantifying the largest connected component size versus fraction of nodes removed.

## Results

### Pipeline design

**Figure 1.**
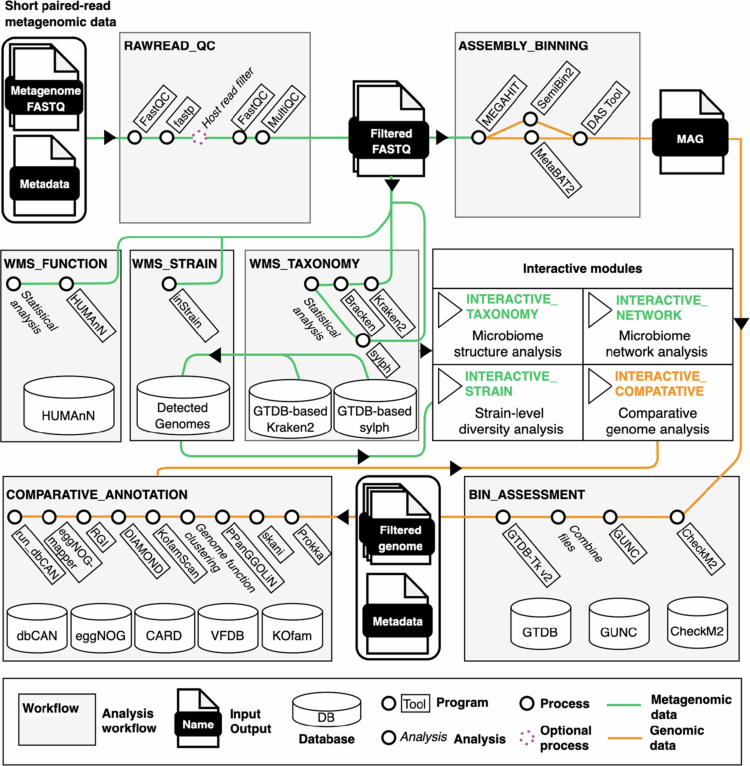
Overview of the metaFun pipeline. The flowchart of metaFun comprises seven analytical modules and four interactive modules. This pipeline allows flexible utilization of each module harnessing modular architecture. Rounded rectangles represent the input data points of users. Inputs are metagenomic reads or metagenome-assembled genomes including respective metadata. Each module generates several outputs. Details are described in the documentation: https://metafun-doc.readthedocs.io/en/latest/index.html.

metaFun integrates analyses of metagenomic reads and MAGs using the GTDB classification system. This workflow consists of seven analytical modules and four interactive analysis modules (Figure 1), processing short metagenomic sequences and assembled genomes with metadata. This pipeline can process quality control of raw metagenomic data, *de novo* assembly, binning, quality assessment and taxonomic assignment of genomes, genomic comparison, taxonomic and functional profiling of metagenomic reads, population microdiversity analysis, and network analysis. Interactive modules were designed for microbiome structure analysis, microbiome network analysis, strain-level diversity analysis, and comparative genomic analysis. All programs were containerized into Apptainer container images. A Conda environment was constructed and deposited into the Bioconda channel^52^ for easy installation and execution of the pipeline. Module components were selected based on performance evaluations in previous benchmark studies, update status, and computational efficiency (Supplementary Note 1 A and Table S1). We evaluated various programs and parameters using simulated data (Table 1), with focus on *de novo* assembly, taxonomic composition profiling of metagenomes, and comparative genomic analysis.

**Table 1.** Information on metagenomic reads and assembled genomes used for performance evaluation of programs with varying parameters.

Module	Step	Value				
ASSEMBLY_BINNING, WMS_TAXONOMY	*De novo* assembly, Binning, Metagenome taxonomic profiling	Data type	Simulated metagenomic reads according to CAMI profiles			
		Environment	Human gut		Marine	Rhizosphere
		Number of samples	5		5	5
		Number of reference genomes	1057		977	894
		Dataset size (Gb)	5			
		Simulator	ART			
		Read length (bp)	150			
		Data repository	https://frl.publisso.de/data/frl:6425518		https://frl.publisso.de/data/frl:6425521	
COMPARATIVE_ANNOTATION	Pangenome and core genome analysis	Data type	Simulated genomes from GTDB release 214			
		Number of genomes	3350			
		Number of species	67			
		Data repository	https://ftp.ncbi.nlm.nih.gov/genomes			
		Genome quality	Complete	Fragmented		Fragmented& incomplete
		Number of samples	3350	3350		3350

### Performance evaluation of *de novo* assembly and binning combinations

MEGAHIT and metaSPAdes are state-of-the-art assemblers. MEGAHIT excelled in high-complexity datasets, while metaSPAdes slightly outperformed MEGAHIT in the CAMI II benchmark across several metrics.^53^ We assessed *de novo* assembly performance using default configuration of metaSPAdes and two configurations of MEGAHIT across three environment using simulated metagenomes (Table 1). Performance varied by environments and assembly configurations (Figure S1 and Table S2). MEGAHIT outperformed metaSPAdes in total length of contigs and fraction of aligned base pairs to reference genomes, especially in complex systems such as the rhizosphere. Meanwhile, metaSPAdes yielded shorter misassembled contig for human gut and marine data. Based on the overall metrics and resource efficiency, MEGAHIT was chosen.

**Figure 2.**
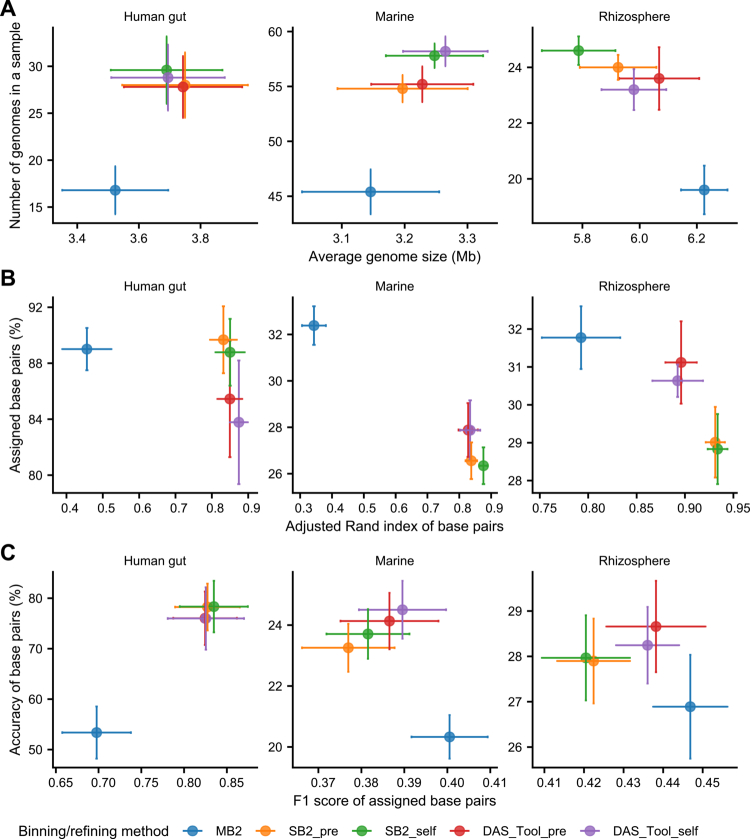
Performance evaluation of the binning/refining combinations on the CAMI data set across three environments. Performance evaluation was conducted using five binning/refining combinations: MetaBat2 (MB2, blue), SemiBin2 using a prebuilt model (SB2_pre, orange), Semibin2 using self-supervised learning (SB2_self, green), DAS Tool combining MB2 and SB2_pre (DAS_Tool_pre, red), and DAS Tool combining MB2 and SB2_self (DAS_Tool_self, purple). All metrics were calculated using AMBER. (A) Average genome size and number of genomes generated in a sample using five binning/refining combinations. All genomes meet the quality threshold of completeness ≥50% and purity ≥90%. (B and C) Performance evaluation of binning/refining combinations using adjusted Rand index, assigned base pairs, F1 score of assigned base pairs, and accuracy of base pairs metrics. Points represent mean values over five samples in each environment, and error bars indicate standard errors.

Generation of accurate MAG is a vital process in genome-resolved metagenomics. MetaBAT2, SemiBin2, and DAS Tool for binning and refinement were selected (Supplementary Note 1E). Five binning/refining combinations were evaluated using multiple metrics: completeness (%) and purity (%) of genomes, adjusted Rand Index (ARI) of base pairs, assigned base pairs (%), F1 score of assigned base pairs, and accuracy of base pairs (%) (Figure 2). MetaBAT2 yielded fewer MAGs than other methods, which produced similar numbers. DAS Tool generated a similar number of MAGs to SemiBin2 with comparable completeness and purity scores (Figure S2). Refinement led to an increase in the average genome size in marine and rhizosphere environments, whereas it remained similar in the human gut (Figure 2A).

We then assessed the sample-wise performance metrics (Figure 2B, C and Table S3). MetaBAT2 utilized more base pairs in marine and rhizosphere samples but showed the lowest ARI. The self-learning mode of SemiBin2 slightly enhanced ARI while marginally reducing assigned base pairs. Refinement improved the accuracy of base pair assignments with a marginal decrease of ARI in marine and rhizosphere samples, whereas human samples exhibited opposite trend (Figure 2C). SemiBin2's self-learning mode enhanced the binning outcomes of complex metagenomes. Consequently, we set the DAS Tool refinement with self-learning mode of SemiBin2 as our default binning method.

### Parameter selection for precise functional annotation and comparative genomic analysis using simulated low-quality genomes

Low genome completeness affects functional annotation accuracy, and core gene threshold selection should be adjusted when using MAGs.^12^^,^^54^ Pangenome structure and analysis are substantially influenced by gene family definition and clustering methodology.^55^ To evaluate this, two genome sets, “Fragmented” and “Fragmented Incomplete”, were generated from 3,350 complete genomes (“Complete”; 67 species, GTDB r214). The completeness of genomes was reduced in both datasets with a further decrease in the “Fragmented Incomplete” dataset (Figure S3A). The “Fragmented” showed more gene families per species than “Complete”. The gene family clustering at 90% identity and 50% coverage consistently increased gene family numbers versus 80% identity 80% coverage (****P < 0.0001; paired *t*-test) (Figure S3B and Table S4).

**Figure 3.**
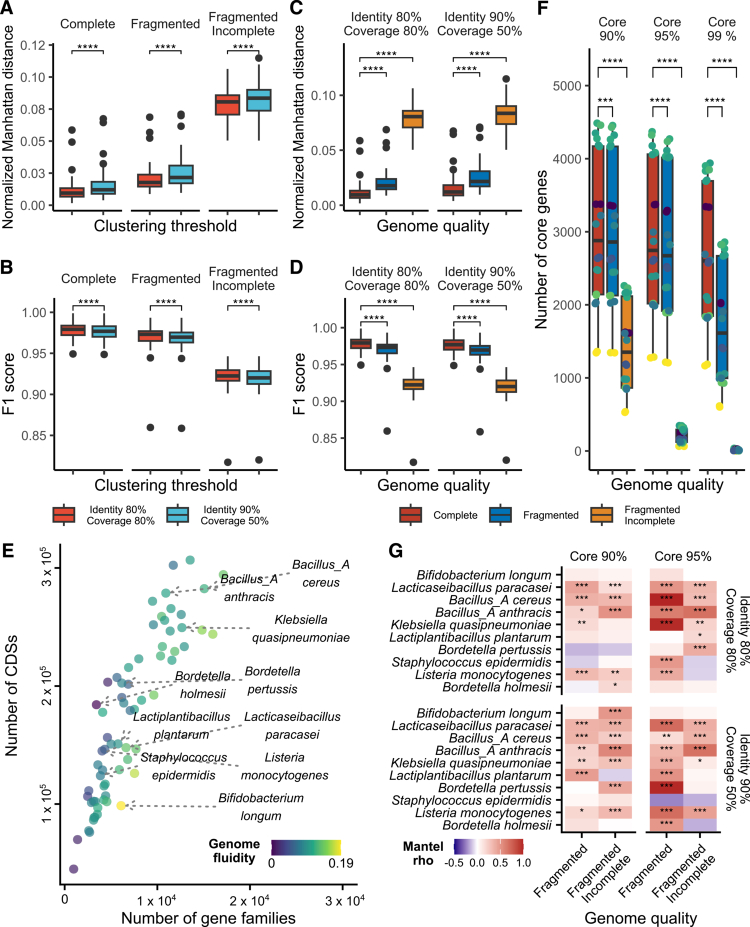
Performance evaluation of parameters for precise comparative genomic analysis using simulated genomes with compromised quality. (A–D) Gene family clustering threshold and genome quality effects on pangenome-based functional annotation accuracy assessed by the normalized Manhattan distance and F1 score. Normalized Manhattan distance represents differently annotated KEGG Orthology annotation divided by the number of total CDSs in a species. F1 score was calculated based on the presence or absence of KO genes within a species, comparing pangenome-based annotations with the individually annotated reference genomes (****P < 0.0001, Wilcoxon signed-rank test). (E) The number of gene families and all Coding sequences (CDSs) of each species. Ten species were selected at regular intervals from the lowest to the highest genome fluidity. (F) Number of core genes for the selected 10 species across three core gene thresholds (90%, 95%, 99%). Box plots are filled with distinct colors based on genome quality corresponding to the color in C and D. (G) Impact of core gene threshold on accuracy of phylogenetic tree inference based on core genome. The species were shown in descending order of genome fluidity. The correlation of phylogenetic trees was calculated using patristic distance matrixes with the Mantel test (Spearman’s rank correlation, 999 permutations, *P < 0.05, **P < 0.01, ***P < 0.001, ****P < 0.0001).

We assessed the impact of genome quality, clustering threshold of gene family, and core gene threshold on downstream analyses. The effects of genome quality and clustering thresholds on functional annotation accuracy were measured using normalized Manhattan distance and F1 score metrics (Table S5). Compromised genome quality significantly reduced functional annotation accuracy (****P < 0.0001, Wilcoxon rank-sum test) (Figure 3A–D), with 90% identity 50% coverage condition yielding less accurate annotation.

For 67 species, we calculated the genome fluidity (Figure 3E), a representative metric for pangenome analysis that measures species' genetic diversity.^56^ A strong negative correlation was observed between F1 score of functional annotation and genome fluidity (*P* < 0.0001, Spearman's rho: −0.81), while correlation between genome fluidity and normalized Manhattan distance was weaker (*P* = 0.035, Spearman's rho: 0.26). Next, we examined the impact of gene family clustering threshold on genome fluidity inference. In “Complete”, genome fluidity did not correlate with gene family numbers (*P* = 0.5556, Spearman's rho: 0.073). Species with low genome fluidity generally showed smaller deviations with 90% identity 50% coverage (Figure S4A). Both low-quality datasets exhibited significant deviations in genome fluidity, as exemplified by *Bordetella holmesii* which increased from 0.002 to 0.148 in “Fragmented Incomplete” (Figure S4A, B). This highlights caution for MAG interpretation, especially for host-adapted species with closed pangenomes.^57^ The absolute and relative differences of genome fluidity in “Fragmented” and “Fragmented Incomplete” were marginally decreased with 90% identity and 50% coverage threshold (Figure S4C, D). Interpretation of pangenome characteristics with incomplete genomes should be carefully conducted.

We quantified genome quality and gene family clustering threshold effects on core genome construction and phylogenetic tree inference. For computational efficiency, we selected ten species out of 67 species at regular intervals of genome fluidity ranging from the lowest to the highest (Figure 3E and Table S6). Core gene numbers significantly decreased with lower genome quality, almost vanishing at 99% threshold in “Fragmented Incomplete” (Figure 3F). For “Fragmented”, a 95% core gene threshold generally produced more accurate phylogenetic topologies than 90% threshold, while results varied for “Fragmented Incomplete” (Figure S5). The 90% identity 50% coverage threshold generally performed well for core genome-based phylogenetic inference, but optimal thresholds were species-specific (Figure 3G).

While the 80% identity 80% coverage yielded more accurate functional annotation, its impact on phylogenetic tree inference was inconsistent. Based on comparisons, we set a 90% core gene threshold and 80% identity 80% coverage as defaults for the COMPARATIVE_ANNOTATION.

### Accurate prokaryotic taxonomic classification by selecting optimal parameters

Precise metagenome interpretation requires distinguishing taxonomic abundance (proportions of microbial taxa) from sequence abundance (proportions of microbial sequences).^58^ Kraken2 with Bracken estimates sequence abundance, while sylph estimates taxonomic abundance. Gold taxonomic profiles were generated for both abundance types, converting NCBI taxonomy to GTDB r220 taxonomy. We compared accuracy of profilers using reference databases constructed with GTDB r220 genomes, evaluating metrics of L1 norm error, Bray-Curtis dissimilarity, and F1 score.

We next investigated the effect of varying parameters. The performance of sequence abundance profiling varied significantly with adjustment of confidence and relative abundance filtration threshold (Figure S6A). Metric values fluctuated by three folds for compositional distance and 45 folds for F1 score. In contrast, sylph showed minimal variation across different compression parameters (Figure S6B) and minimum *k*-mer counts (Figure S7 and Table S7). Taxonomic coverage of sylph's database exerted minimal influence, whereas unknown estimation option decreased accuracy and poorly estimated unknown sequence proportions (Figure S8).

**Figure 4.**
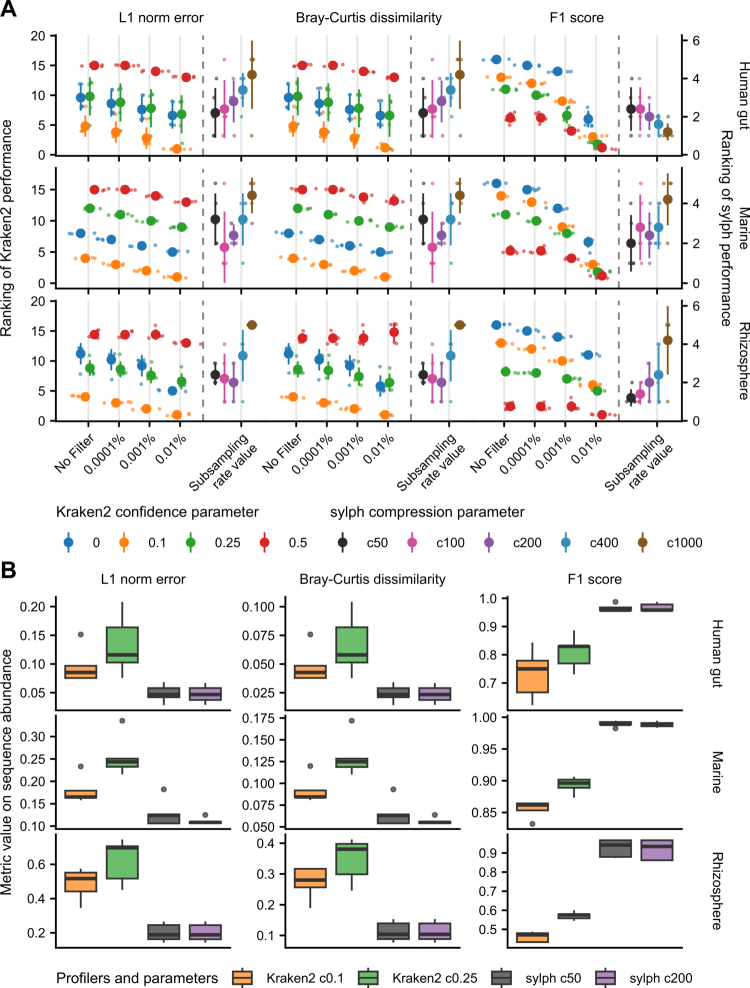
Performance evaluation of the profiling parameters of Kraken2 with Bracken, and sylph for accurate taxonomic profiling. Effects of the varying confidence threshold of Kraken2, relative abundance filtration of Bracken, and compression value of sylph on taxonomic profiling accuracy were evaluated. Kraken2 in the figure represents the taxonomic profiling results of Kraken2 with Bracken. (A) Comparison of taxonomic classification performance using varying Kraken2 confidence levels (0–0.5) with different relative abundance filtration thresholds (0%–0.01%) and sylph compression parameters (c50–c1000). L1 norm error, Bray-Curtis dissimilarity, and F1 score were evaluated across the human gut, marine, and rhizosphere environments (*n* = 5 each). Taxonomic profiles were generated with the GTDB r220 database and compared to sequence abundance gold taxonomic profiles. Large points represent the mean ranking value of the parameter condition, and small points represent the ranking value of an individual sample. Performance of Kraken2 with Bracken and sylph is ranked independently and separated by the dashed line. Two tools have respective y-axis (left, Kraken2; right, sylph). (B) Performance evaluation of optimal parameters for Kraken2 with Bracken (confidence levels 0.1 and 0.25) and sylph (compression parameters c50 and c200) across the three environments. Box plots show the distribution of metric values for selected profiler-parameter combinations.

In taxonomic abundance profiling, Kraken2 with 0.1 confidence threshold performed best for taxonomic composition distance, while 0.5 confidence showed robust F1 scores with poor composition estimation. Higher Bracken filtration thresholds generally yielded better accuracy. Lower sylph compression value did not consistently enhance profiling accuracy. After ranking conditions, (Figure 4A) we selected confidence values of 0.1 and 0.25 with filter threshold 0.01% for Kraken2 with Bracken and c50 and c200 for sylph. Although the Kraken2 with Bracken database was not optimized for specific environments, optimized parameters demonstrated reliable accuracy in taxonomic profiling.

In taxonomic accuracy evaluations, sylph generally outperformed Kraken2 with Bracken with evaluated metrics with computational efficiency (Figure 4B). While sylph cannot classify individual reads, Kraken2 can classify taxonomy of individual reads. Therefore, metaFun provides both methods: Kraken2 with Bracken for sequence abundance profiling and sylph for taxonomic abundance profiling using GTDB r220 database.

### Pipeline implementation

We integrated analytical modules and interactive analysis modules into a pipeline with configurable parameters (Figure 1). Following FAIR principles, program environments were packaged into Docker (https://www.docker.com) images and converted into Apptainer (https://github.com/apptainer/apptainer) images to prevent dependency conflicts. The execution environment was packaged into a Conda environment and deposited into Bioconda repository (https://anaconda.org/bioconda/metafun). Analytical modules were generated using Nextflow.^59^ Source codes and modules are accessible under the MIT license via https://github.com/aababc1/metaFun. Databases are available at https://www.microbiome.re.kr/home_design/Database.html, downloadable via metaFun.

We designed seven analytical modules and four interactive modules for modular use: RAWREAD_QC, WMS_TAXONOMY, WMS_FUNCTION, WMS_STRAIN, ASSEMBLY_BINNING, BIN_ASSESSMENT, COMPARATIVE_ANNOTATION, INTERACTIVE_TAXONOMY, INTERACTIVE_NETWORK, INTERACTIVE_STRAIN, and INTERACTIVE_COMPARATIVE. Documentation with detailed explanations and examples is at https://metafun-doc.readthedocs.io/en/latest/index.html.

#### RAWREAD_QC module—Quality control of metagenomic reads

This module uses fastp^60^ for adapter removal and quality control of metagenomic reads, optional host read removal using Bowtie2.^61^ FastQC (https://www.bioinformatics.babraham.ac.uk/projects/fastqc) and MultiQC^62^ provide read-quality assessment and statistics. The outputs are utilized for downstream modules.

#### WMS_TAXONOMY module—Taxonomic profiling of metagenomic reads

Kraken2 with Bracken and sylph are used with GTDB r220 database for prokaryotes profiling. Based on our evaluation, default parameters were set (0.1 confidence for Kraken2, 0.01% filtration for Bracken, c200 for sylph). Profiling results are packaged into a phyloseq object^63^ for use in the interactive WMS_TAXONOMY module.

#### INTERACTIVE_TAXONOMY module—Interactive analysis of taxonomic composition of metagenome

An interactive shiny application allows rapid exploratory data analysis, including alpha and beta diversity, metadata distribution, and differential abundance analysis via integrated MaAsLin2^64^ with various abundance transformation models and taxonomic ranks.

#### WMS_FUNCTION module—Functional profiling of metagenomic reads

HUMAnN3^65^ performs functional annotation of quality-controlled reads. Diversity metrics and static figures are generated using superClass and pathway information of MetaCyc^66^ with provided metadata.

#### WMS_STRAIN module—Population microdiversity assessment of metagenome

This module performs strain-level microdiversity analysis using taxonomic profiles from the WMS_TAXONOMY module. Reference genomes from GTDB are automatically selected based on detected taxa to build database. inStrain^46^ performs read mapping for population microdiversity profiling.

#### INTERACTIVE_STRAIN module—Interactive analysis of population microdiversity of metagenome

Users can interactively inspect population microdiversity of genomes and genes. Strain sharing analysis, microdiversity metrics including nucleotide diversity, SNV density, pN/pS, and population-level ANI can be interactively visualized per genome and gene.

#### ASSEMBLY_BINNING module—De novo assembly of metagenomic reads and binning

Quality-controlled reads are assembled into contigs via MEGAHIT. For complex metagenomes (e.g., soil), usage of the option '*--megahit_presets meta-large*' is recommended following evaluation results. Contigs are renamed with metagenome accession. Reads are mapped to contigs using Bowtie2 and sorted with SAMtools.^67^ MetaBAT2 and SemiBin2 are used for binning and bins are refined and combined using DAS Tool. For complex metagenomes, the use of '*--semibin2_mode self*' is recommended.

#### BIN_ASSESSMENT module—Quality control and taxonomic assignment of MAGs

CheckM2^68^ and GUNC^69^ assess quality of genomes. Genomes passing the selected quality threshold undergo taxonomic classification using GTDB-Tk^70^ with GTDB database. The statistics of genomes can be visually explored with GENOME_SELECTOR in metaFun, with selected genomes proceeding to comparative genomic analysis.

#### COMPARATIVE_ANNOTATION module—Comparative genomic analysis and annotation

This module performs species-level comparative genomic analysis. Prokka predicts CDSs and PPanGGOLiN serves as the pangenome analysis chassis. Representative pangenome sequences are functionally annotated using evolutionary genealogy of genes: Non-supervised Orthologous Groups (eggNOG),^71^ HMM database of KEGG Orthologs (KOfam),^38^ Comprehensive Antibiotic Resistance Database (CARD),^72^ Virulence Factor Database (VFDB),^73^ and Carbohydrate-Active Enzymes (CAZy)^74^ databases. Subspecies-level clustering is executed with default option using skani^75^ of dRep^76^ following 99.5% ANI clustering criteria.^77^

#### INTERACTIVE_COMPARATIVE module—Interactive analysis and exploration of pangenome functional annotation result.

Heatmaps of functional annotations, gene information tables, metadata exploration, PCoA of genome clustering, sequence family finder, gene tree generator, and association analysis between genes and metadata are available in interactive app. It utilizes ComplexHeatmap^78^ and InteractiveComplexHeatmap^79^ for heatmap generation, ggkegg^80^ for KEGG module completeness calculation. Association analyses employ Fisher's exact test for categorical metadata and pyseer^81^ for numerical metadata. MAFFT^82^ and FastTree2^39^ are used for phylogenetic tree and ggtree^83^ is utilized for interactive tree visualization.

### Interactive modules for exploratory data analyses

**Figure 5.**
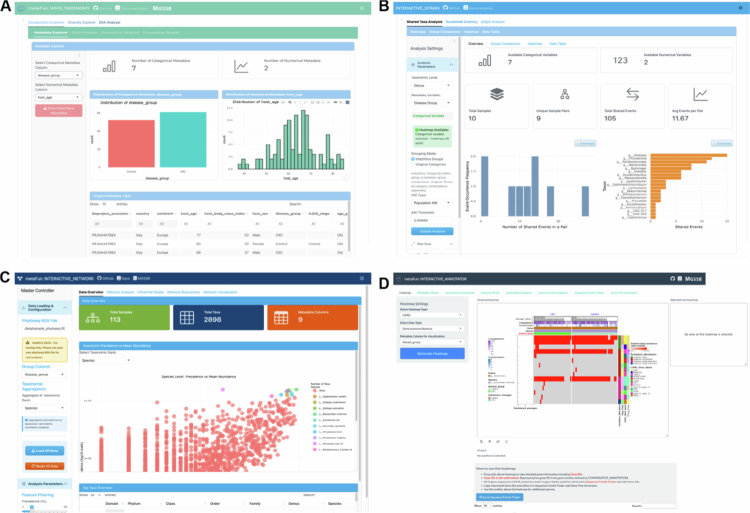
Interactive visualization and analysis modules for comparative taxonomic analysis of metagenomes and assembled genomes. (A) The INTERACTIVE_TAXONOMY module interface for metagenomic taxonomic composition analysis, featuring an interactive composition explorer with customizable visualization options. The interface includes controls for taxonomic rank selection, data filtering, and plotting parameters, with a stacked bar plot showing the relative abundances of different phyla across sample groups. (B) The INTERACTIVE_STRAIN module interface for population microdiversity analysis, showing strain sharing analysis with taxonomic level and metadata variable selection. Users can set ANI thresholds and coverage thresholds for species detection across samples. (C) The INTERACTIVE_NETWORK module interface for microbial co-occurrence network analysis, displaying taxonomic prevalence versus mean abundance plots with feature filtering options for network construction. (D) INTERACTIVE_COMPARATIVE module interface. Many for comparative genomic analysis, showing the interface with multiple analysis tabs including heatmap visualization, metadata analysis, and a volcano plot with associated data table for examining differential gene associations. Users can select metadata columns, adjust visualization parameters, and perform sequence family analysis.

We developed shiny-based interactive apps for structure, network, and population microdiversity analysis of metagenomes, and comparative genomic analysis. These modules facilitate several statistical analyses between genomic information and metadata variables of interest (e.g., host age and health status). We referred established microbiome analysis and visualization practices such as MicrobiomeStatPlots^84^ and designed interactive applications suited for accessible data exploration. The INTERACTIVE_TAXONOMY interactive module supports visualizing single taxon abundance with metadata, taxonomic composition bar and line plots, alpha diversity, beta diversity (Aitchison, Jaccard, and Euclidean distances) using Permutational Multivariate Analysis of Variance (PERMANOVA), and differential abundance analysis using MaAsLin2 (Figure 5A). It accepts phyloseq objects from profiling results from WMS_TAXONOMY. Users can apply transformations (centered log-ratio, log), construct linear models across taxonomic ranks, and customize visualizations (Figure S9).

The INTERACTIVE_STRAIN module supports population microdiversity analysis using inStrain outputs (Figure 5B). Users can visualize nucleotide diversity (*π*), SNV density, and pN/pS ratios at genome and gene levels, with statistical comparisons across metadata groups. Strain sharing analysis based on population ANI enables identification of shared strains across samples.

The INTERACTIVE_NETWORK module enables co-occurrence network analysis and visualization (Figure 5C). Users can infer microbial associations through co-occurrence network construction using FlashWeave or FastSpar, compare network topologies between sample groups, and identify keystone taxa through Zi-Pi classification and centrality metrics. Network robustness can be evaluated through systematic node removal simulations.

The INTERACTIVE_COMPARATIVE module enables visualizing functional annotations (KEGG, VFDB, CARD, CAZy), statistical association analysis of genes with metadata, sequence family search, and genome functional clustering (Figure 5D). Users can select specific metadata variables and thresholds for immediate visualization and statistical analyses (Figure S10). These real-time analysis modules enable rapid hypothesis generation by identifying relevant genetic elements and metadata, enabling comprehensive analysis of species genomes.

### Case application of metaFun to a real metagenomic dataset

**Figure 6.**
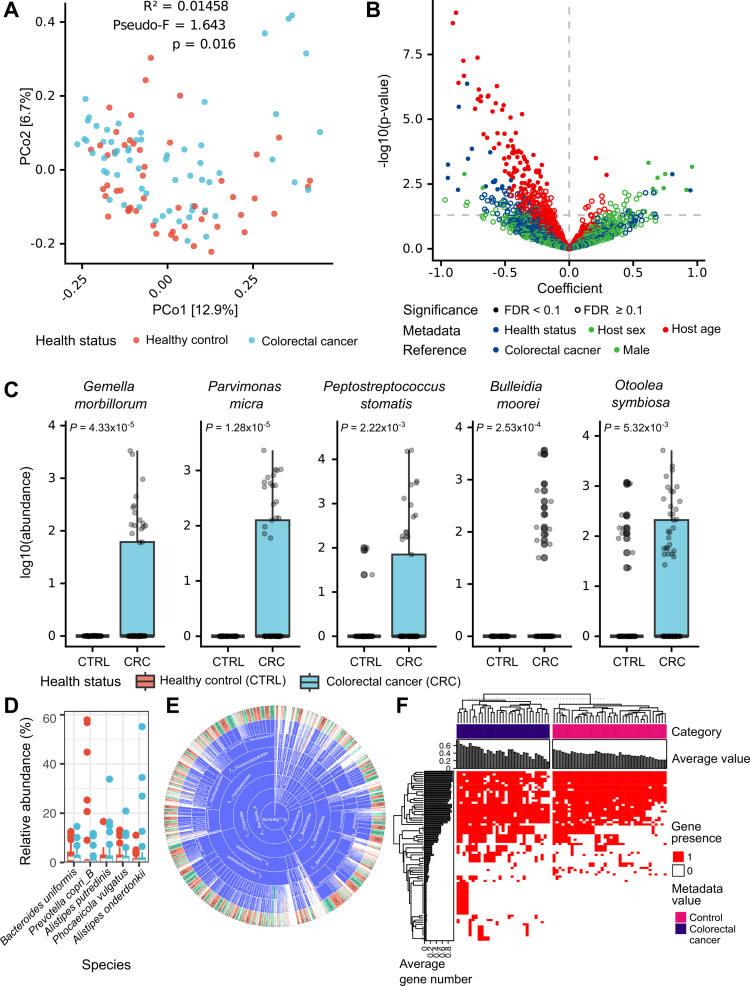
Case metagenomic dataset analysis using the metaFun pipeline. (A) PCoA analysis result using the Bray-Curtis dissimilarity index. The effect of health status on community composition between groups were assessed using permutational multivariate analysis of variance (PERMANOVA). (B) Association analysis result of linear mixed effects model using log_10_ transformed data in interactive module. Health status, age, and sex were set as fixed effects for linear mixed effects model construction. Coefficient value (x-axis) of categorical metadata denotes the contrast between any value and reference value of the selected variable, and the positive coefficient value of numerical variable indicates positive association between taxa abundance and numerical variable. *P* values (y-axis) were determined by a constructed linear model and adjusted for multiple testing using the Benjamini-Hochberg procedure to control false discovery rate (FDR *Q* values). The selected area represents five taxa most significantly associated with colorectal cancer (CRC). (C) Log transformed abundance of the five most significantly associated taxa with CRC. *P* values above box plots represents calculated using Wilcoxon rank-sum test. (D) Relative abundance of the most abundant species grouped by metadata. (E) Sunburst chart in genome selector for the COMPARATIVE_ANNOTATION module. Genome selector visualizes completeness and contamination scores of genomes with scatter plot and shows the distribution of genome numbers according to metadata variables with bar charts. The circular sunburst chart visualizes the number of genomes according to metadata variables at any taxonomic rank. A demonstration case of this application is available at https://www.microbiome.re.kr/dash. (F) The association analysis result of Fisher's exact test using health status as metadata of interest in the interactive module. All significantly associated genes among 69 *Bacteroides uniformis* strains are shown with *Q* value < 0.1 corrected by the Benjamini-Hochberg procedure.

We analyzed a public metagenomic dataset (519 Gb) from 52 CTRL and 61 CRC fecal samples (Table S8) using RAWREAD_QC and WMS_TAXONOMY. While alpha diversity showed no significant difference (Table S9), intra-group Aitchison distance (*P* < 2.2 × 10^−16^, Wilcoxon rank-sum test) and species-level composition (*P* = 0.016, PERMANOVA) significantly differed (Figure 6A). Using linear model with covariates included, we identified 19 species associated with disease status, 9 with sex, and 85 with age (*Q* < 0.1) (Figure 6B and Table S10).

Top CRC-enriched species were *Gemella morbillorum*, *Peptostreptococcus stomatis*, *Parvimonas micra*, *Bulleidia moorei,* and *Otoolea symbiosa* in GTDB taxonomy. Abundances of five taxa were assessed via Taxon Analyzer in interactive module (Wilcoxon rank-sum test) (Figure 6C). *G*. *morbillorum* is a CRC biomarker^85^ and a late-CRC-associated bacteria.^86^ *P. stomatis* accelerated colonic tumorigenesis in mice.^87^ The roles of *P. micra* in CRC development are supported by multi-omics^88^ and *in vivo*^89^ studies. *B. moorei* (*Solobacterium moorei* in NCBI taxonomy) induces inflammation *in vitro* or *in vivo* and is CRC-associated.^90^ *O. symbiosa* (*Clostridium symbiosum* in NCBI taxonomy) showed higher significance than *Fusobacterium nucleatum* in CRC detection and good early CRC detection,^91^ and promoted colorectal tumorigenesis via host cholesterol regulation.^92^ The interactive module facilitated identifying these associations while controlling for confounding factors.

We generated 2,776 MAGs (1,291 from CTRL and 1,485 from CRC) using ASSEMBLY_BINNING and selected *Bacteroides uniformis* with 69 MAGs for comparative genomic analysis with GENOME_SELECTOR (Figure 6D, E). Though typically commensal,^93^ *B. uniformis* has a potential association with ulcerative colitis^94^ and advanced adenoma.^95^ Using COMPARATIVE_ANNOTATION and its interactive module, we identified CRC-associated genes with gene presence/absence heatmap (Figure 6F). Fisher's exact test with FDR correction identified significantly differential gene distributions (*P* < 0.05), with distinct clustering patterns between CRC and CTRL. We annotated function of CRC-associated genes functions using Gene Function Explorer in the interactive module. Of 28 genes uniquely present in MAGs from CRC, 23 were functionally annotated (Table S11), primarily as transposon-related or unknown orthologs, suggesting potential gene introduction from near species.

We applied WMS_STRAIN and INTERACTIVE_STRAIN to examine population microdiversity in the CRC cohort (Figure S11). *Streptococcus, Faecalibacterium, *CAG-103*,* and CAG-217 exhibited significantly elevated SNV density in CTRL compared to CRC. Except for *Streptococcus*, these genera showed concordant trends in nucleotide diversity, suggesting consistent disease-associated changes in population microdiversity. The pN/pS ratio in gene-level analysis revealed that genes involved in replication/recombination (COG category L) and transcription (COG category K) exhibited significant differences between disease states within *Bacillota*_A, *Bacteroidota*, and *Pseudomonadota,* suggesting disease-associated selective pressures on core cellular machinery.

Network analysis using INTERACTIVE_NETWORK revealed substantial topological differences between conditions (Figure S12). The CRC network displayed higher connectivity with increased edge count and density, while modularity was reduced, indicating less compartmentalized community organization in CRC. Network robustness analysis showed increased vulnerability to targeted attacks in CTRL. High-centrality nodes differed distinctly between conditions, with pronounced shifts in taxonomic composition, reflecting disease-associated reorganization of microbial interaction structures.

This analysis demonstrates how metaFun integrates read-level analysis, MAG generation, comparative genomic analysis, strain-level metagenomic analysis, network analysis, and multiple interactive analyses, enabling efficient processing from raw data to statistical analysis with neat visualization. The streamlined workflow significantly reduces the complexity of conducting multi-step analyses, allowing rapid identification of metadata-genetic feature associations in microbial populations.

## Discussion

metaFun is engineered to handle large-scale metagenomic analysis. Its architecture, built using Nextflow and Apptainer, facilitates the processing of massive datasets by employing parallel computing and containerization for efficient workflow. By comparing previous studies and evaluating parameters, we set a standard operating procedure to maintain analysis accuracy and implement it into the metaFun pipeline. metaFun works in Linux-based environment and all modules were containerized to ensure consistent results. The modular architecture enables users to add high quality genomes or metagenomes to expand analysis scale.

metaFun differs from previous MAG pipelines like ATLAS, nf-core/mag, MAGNETO, and Metaphor. For the assembly method, we adopted single-sample assembly and single-sample binning whereas nf-core/mag, MAGNETO, and Metaphor adopted co-assembly, which is not scalable to hundreds of samples, risks chimeric sequence incorporation, and induces subspecies-specific contig loss.^96^^,^^97^ For taxonomic profiling, ATLAS utilizes generated MAGs as database, and Metaphor annotates contig taxonomy using COG NCBI taxonomy information. In contrast other tools utilize specialized taxonomic profilers. The minimum coverage at which MAG could be recovered was at least 3–4 folds of genome length,^98^ and insufficient database coverage may introduce biases. In metaFun, Kraken2 with Bracken and sylph were utilized with optimized parameters. We observed that a broad taxonomic coverage database based on GTDB r220 could accurately profile taxonomy composition. In addition to MAG generation and quality assessment, metaFun provides modules for comparative genomic analysis, facilitating interactive analysis at species-level. We automated most steps required in metagenome analysis to minimize human errors. We followed database-specific ontologies established by experts such as CARD, VFDB, eggNOG, and KO. The most distinguished feature of our pipeline is dynamic interactive analysis modules for code-free and rapid exploratory data analysis. We developed two interactive modules that can interactively visualize and analyze results of taxonomic composition and comparative genomic analysis.

Our case application of metaFun revealed taxa significantly associated with CRC. Recent studies have emphasized that proper data transformation and control of covariates increase the accuracy of differential abundance analysis.^99^^,^^100^ The integration of MaAsLin2 in the metaFun interactive module identified CRC-associated taxa using a linear model adjusted for demographic variables. Five species with the highest statistical significance emerged as pro-inflammatory microbes in the gut environment. We also identified two *Fusobacterium* species in GTDB taxonomy, *F. polymorphum*, *and F. animalis*. *F. nucleatum*, previously recognized as a CRC biomarker,^101^ was proposed to be reclassified into several species in NCBI taxonomic criteria^102^ and divided into several species in GTDB taxonomy. In recent studies, clade- or subspecies-specific associations with disease status have been reported using pangenome-metagenome combined analyses (*F. nucleatum* subspecies *animalis*^103^ and *F. nucleatum* subspecies *polymorphum*^104^ in NCBI taxonomy criteria). This result demonstrates that the usage of linear model benefits from controlling spurious associations by accounting for confounding factors. We generated MAGs using the ASSEMBLY_BINNING module and evaluated their quality with the BIN_ASSESSMENT and the genome selector. Using the result of the COMPARATIVE_ANNOTATION and its interactive module, we could readily identify metadata-associated genes within the module. The species-level pangenome and genome dataset could be easily constructed and annotated for downstream analyses using our pipeline. For instance, such constructed pangenomes could be applied to metagenomic read mapping for subspecies-level genomic analysis.

In our CRC cohort analysis, strain-level profiling revealed elevated SNV density and nucleotide diversity in a specific genus including *Faecalibacterium*, CAG-103, CAG-217, among CTRL samples, suggesting disease-associated decreases in population heterogeneity. The concordant trends between SNV density and nucleotide diversity metrics reinforce the biological relevance of these observations.^105^^,^^106^ Furthermore, functional category-specific pN/pS analysis identified selective pressures on genes involved in replication and transcription machinery, potentially reflecting adaptive responses to the altered gut environment in CRC. It is well known that environmental effects on microbial population differ across species,^107^ and this module enables accurate quantification of microdiversity at the gene level, tailored to each species in response to specific environmental conditions. Network topology analysis demonstrated increased connectivity and reduced modularity in CRC associated microbial communities, with distinct hub taxa between conditions.^108^ These patterns align with prior observations of network characteristics comparison.^109^ Notably, networks from healthy individuals exhibited a higher proportion of negative correlations, consistent with established models positing that a balanced fraction of antagonistic interactions sustains microbial community stability.^110^ Targeted removal of high-centrality nodes in healthy networks led to a rapid decline in the largest connected component, underscoring the pivotal role of these central hubs in maintaining overall network integrity.^111^

The integration of INTERACTIVE_STRAIN and INTERACTIVE_NETWORK modules extends metaFun's analytical scope beyond compositional profiling. The WMS_STRAIN module, built upon inStrain, enables population-level genetic variation assessment directly from metagenomic reads, while the network module facilitates ecological interaction inference from co-occurrence patterns. Importantly, these modules are seamlessly connected to upstream outputs enabling immediate exploration of community-level interaction structures.

The current version of metaFun focuses on short-read metagenomic data analysis, which demonstrates the highest accuracy and widespread utility in the field. Especially the modular architecture of metaFun will benefit from component upgrades and exchanges. Further, the more scalable analysis methods will be tested for rapid and accurate analysis and integrated into metaFun. We employed deep learning–based SemiBin2 for binning, which generated highly accurate MAGs from soil samples used to investigate responses to global change factors.^112^ In the next version of our pipeline, we plan to integrate additional deep learning–based methods to further enhance binning, taxonomic classification, and functional annotation performances, as demonstrated by recent advances.^113^ Beyond single-domain analysis, we plan to extend metaFun to support multi-domain metagenomic data encompassing bacteria, archaea, fungi, and viruses. This extension will require integration of scalable processing modules optimized for the increased computational demands of cross-domain analyses. Additionally, we aim to incorporate inter-domain network analysis capabilities using tools such as ggClusterNet2,^114^ which enables detection of cross-kingdom microbial interactions and co-occurrence patterns. We believe that metaFun facilitates large-scale metagenomic data analyses with both scalability and accuracy through its user-friendly features, including interactive analysis modules, comprehensive result visualization, and semi-automated data transformation.

## Conclusions

metaFun generates taxonomic and functional profiles as well as MAGs from metagenomic read data. It performs comparative genomic and metagenomic data analyses. This pipeline has convenient functionality such as host genome indexing, interactive MAG statistics visualization and selection, interactive apps using metagenome taxonomic profiles and results of comparative genomic analyses. The main strength of metaFun lies in seamless integration of data generation with results analysis and visualization using standardized environments. It will foster standardized metagenomic data analysis with easy implementation and result analyses. Future updates will focus on scalable analysis of large-scale metagenomic data.

## Supplementary Material

metaFun_supplementary_materials.docx metaFun_supplementary_materials.docx

metaFun_supplementary_shortened_new.xlsx metaFun_supplementary_shortened_new.xlsx

## Data Availability

Taxonomic profiles and modified taxonomy information for metagenome simulation, binning performance evaluation metrics with AMBER, 3,350 complete genome information, accuracy metrics of varying parameters of Kraken2 with Bracken and sylph, and MAG statistics generated by metaFun are deposited at https://doi.org/10.17605/osf.io/xg9dj. The source code of metaFun is available at https://github.com/aababc1/metaFun under the MIT license. Detailed documentation for the execution of this pipeline is available in supplementary information and at https://metafun-doc.readthedocs.io/en/latest. The databases and case rendered apps of this pipeline are accessible at https://www.microbiome.re.kr/home_design/Database.html.
