# Supplementary material for: metaFun: An analysis pipeline for metagenomic big data with fast and unified functional searches
Source: Gut Microbes. 2026 Jan 13;18(1):2611544. doi: 10.1080/19490976.2025.2611544 (PMC12818822; doi:10.1080/19490976.2025.2611544)
Supplement: metaFun_supplementary_materials.docx [file KGMI_A_2611544_SM3881.docx]

**metaFun: An analysis pipeline for metagenomic big data with fast and unified functional searches**

Hyeon Gwon Lee^1^, Ju Yeon Song^1^, Jaekyung Yoon^1^, Yusook Chung^2^, Soon-Kyeong Kwon^3^ and Jihyun F. Kim^1,4,*^

**Supplementary Notes and Supplementary Figures**

**Supplementary Note 1: Evaluation of programs and databases for pipeline incorporation**

**Supplementary Note 1A. Program and database selection criteria**

We aimed to construct an accurate and scalable pipeline for metagenomic data analyses. Recent advances in metagenomic sequencing have promoted the development of sequence analysis programs. Metagenomic data analysis typically involves two approaches: read-based analysis and assembly-based analysis [1, 2]. Assembly-based analysis enables information retrieval of novel and uncultivated microorganisms, while read-based analysis enables read mapping analysis and community-level analysis, including the detection of low-abundance taxa. These two approaches should be combined to fully understand the genomic entities of metagenomic data.

For pipeline design and program selection, we considered several components: regular update status, performance, speed, and executability. Researchers often choose famous programs in terms of citation score, journal impact factor, and author reputation. However, one study revealed that these factors do not necessarily correlate with program performance, and speed and accuracy of program were not inversely correlated as commonly believed. Instead, regular update status was associated with program accuracy and performance [3]. Hence, programs and databases with sustained updates were prioritized for integration into the pipeline. Our selection process referenced established best practices [4], standard operating protocols [5], and web-based platforms [6] for metagenomic data analysis pipeline design. For accurate analysis, proper raw data preprocessing and reproducible and standardized protocols are imperative. Furthermore, we reviewed previous benchmark studies to identify top-performing methods for pipeline integration. We evaluated various programs and parameters following recent recommendations to find optimal components [7].

**Supplementary Note 1B. Selection of components for the RAWREAD_QC module**

Sequencing reads contain sequencing error, adaptor sequences, and host contamination, all of which should be addressed for reliable metagenomic data analysis. Raw sequences from Illumina sequencers exhibit median error rates ranging from 0.08% to 0.6% [8]. Inadequate quality control of reads led to error-prone results during read mapping, *de* *novo* assembly, and variant calling processes [9]. The sensitivity of taxonomic profiling diminishes without host sequence decontamination [10]. Hence, adapter sequence removal, quality control, host sequence decontamination, and quality assessment of reads should be performed in this step.

fastp [11], Cutadapt [12], and Trimmomatic [13] are frequently utilized for read preprocessing. Cutadapt and Trimmomatic are state-of-the-art tools, but adaptor sequence information should be provided for operation, while fastp provided additional functionality and decent performance compared to the two programs in read quality statistics [14]. It automatically detects and removes primer sequences, and trims reads based on quality scores. In addition, additional functionality such as deduplication and faster speed than other programs are strengths of fastp. For these reasons, fastp was chosen as the sequence preprocessing tool for metaFun.

To remove the host genome sequences, we reviewed previous benchmark studies of read alignment. Bowtie2 [15] and BWA-mem [16] have been widely utilized for reference read mapping. Bowtie2 showed remarkable performance in accuracy and speed [17, 18]. We thus selected Bowtie2 for host read detection and removal from metagenomic data.

FastQC (https://www.bioinformatics.babraham.ac.uk/projects/fastqc/) is a standard tool inspecting various quality statistics of reads and MultiQC [19] aggregates read quality reports of FastQC from both raw and processed read results. Two tools were selected as our read quality assessment tools.

**Supplementary Note 1C. Selection of components for WMS_TAXONOMY module**

Taxonomic composition profiling via read-based methods is a standard procedure to characterize microbial information in the metagenomic data. This could be executed by one of DNA-to-DNA, DNA-to-protein, or marker-based classification methods [20]. The performances of taxonomic profiling are evaluated by accuracy metrics (sensitivity, precision, and F1 score) alongside taxonomic composition distances (L1 norm error and Bray-Curtis dissimilarity). Renowned programs are MetaPhlAn [21] and mOTUs [22] for marker-based classification, Kraken2 [23] and Bracken [24] for DNA-to-DNA, and Kaiju [25] and MMseqs2 [26] for DNA-to-protein classification. In a 2019 benchmark study, DNA-to-DNA profilers (e.g., Kraken2 and Bracken) showed strong precision and recall with a requirement of high memory volume, whereas marker-based profilers efficiently utilized memory and CPU [27]. In Critical Assessment of Metagenome Interpretation (CAMI) II [28], mOTUs v.2.5.1 and MetaPhlAn v.2.9.22 ranked best in sensitivity and precision, and mOTUs, MetaPhlAn and Bracken v.2.6 showed top-tier performance in taxonomic composition profiling. Only Bracken and Metalign [29] detected viruses in the CAMI II challenge. Kraken2 and MetaPhlAn are now recognized as state-of-the-art programs for taxonomic composition analysis and have proven to show top-tier performance.

Kraken2 showed a higher proportion of false positive taxa than MetaPhlAn4 in some studies [30, 31]. Proper adjustment of Kraken2 parameters is essential, and it significantly affects the accuracy of profiling results [32, 33]. Furthermore, novel microbial taxa cannot be profiled using prebuilt databases, and the creation of a new database including novel genomes should be executed for accurate taxonomic composition profiling [34]. Recently, an average nucleotide identity (ANI)-based taxa detection method was developed that exhibited better performance in accuracy metrics and showed exceptional efficiency in resource usage [35].

Considering database modifiability for performance evaluation analysis, profiling accuracy, and computing resource efficiency, we selected Kraken2 with Bracken and sylph, as taxonomic composition profilers. MetaPhlAn showed remarkable performance, but we decided not to include this tool in our pipeline. The database of MetaPhlAn was built based on more than one million genomes, and the information of genomes is not fully disclosed. The addition of clade-specific marker genes requires expertise in microbial genomics, and it is difficult to modify the database for users. Consequently, we evaluated the accuracy of Kraken2 with Bracken and sylph with varying parameters using the Genome Taxonomy Database (GTDB) [36] r220 database.

**Supplementary Note 1D. Selection of components for WMS_FUNCTION module**

Functional annotation of metagenomic data can be categorized into two approaches. The first approach is to *de novo* assemble metagenomic reads, construct and annotate a gene catalog, and map reads to the gene catalog. The second approach is to map metagenomic reads directly to prebuilt sequence databases with functional annotations. Gene catalog-based metagenome annotation is widely adopted for functional annotation of metagenomic data in multiple analysis pipelines [37, 38]. However, generating a gene catalog for a specific dataset requires careful consideration. For instance, sequence identity-based metagenomic clustering can contain transitive clustering error, taxonomic inconsistency, and false positive mapping to other genes [39]. While gene catalog generation could be beneficial for capturing novel genomic information, we did not include gene catalog construction in our pipeline due to the aforementioned issues. For the metaFun pipeline, HUMAnN3 [40] was integrated as our functional annotation method for metagenomic data, owing to well-curated metabolic terminology and its ability to generate reliable results, despite requiring a modest level of computational resources.

**Supplementary Note 1E. Selection of components for ASSEMBLY_BINNING module**

*De novo* assembly is an essential aspect of assembly-based metagenomic data analysis. For assembly-methods, co-assembly and single-sample assembly could be utilized. The co-assembly strategy tends to generate longer and less fragmented contigs, and possibly generate low-abundance species contigs. However, co-assembly could obscure subspecies-level genetic variations [41, 42], and computational resource usage increases along with the increase in sample number [43]. One of our pipeline purposes is scalability, therefore we decided to adopt a single-sample assembly strategy in this version.

Numerous benchmark studies have evaluated the performance of *de novo* assemblers for metagenomic data [44, 45]. Among the assemblers, MEGAHIT [46] and metaSPAdes [47] are frequently reported to exhibit robust and top-tier performance. In early benchmark results, metaSPAdes outperformed MEGAHIT [48], but more recent research has found that the newer version of MEGAHIT surpasses metaSPAdes in many metrics. For instance, MEGAHIT v.1.2.9 showed better assembly statistics in deep sequencing depth (>100X) than metaSPAdes v.3.15.0 [45] and vice versa. In strain-resolved viral genomes and microbial 16S rRNAs, MEGAHIT v.1.2.9 speed was significantly faster, performed well at lower genome coverage, and assembled a higher fraction in strain-level than metaSPAdes v3.15.2 [49]. Given that assembler performance varies by program version and analysis environment, we decided to compare the performance of MEGAHIT and metaSPAdes. It is worth noting that some genetic elements, such as ribosomal genes [50], mobile genetic elements [51], and antimicrobial resistance genes [52], are technical challenges to be assembled in proper genomic contexts.

A metagenome-assembled genome (MAG) is generated by binning, which assembles contigs using quality-controlled metagenomic reads. Binning methods have undergone significant advances through algorithm developments. The nucleotide composition, abundance, and graph structure of sequences are utilized to cluster sequences [53]. Recently, deep-learning-based algorithms have been adopted for the binning process, such as VAMB [54] and SemiBin2 [55], which showed improved binning performance. Nevertheless, systematic evaluation of binning methods with multiple metagenomic data, comprising a wide range of species and strain diversity across various environments, revealed no single binning method is universally optimal [28].

Two binning strategies can be applied to the binning process: single-sample binning and multi-sample binning, both following single-sample assembly. Multi-sample binning is likely to contain less contamination according to recent research [56]. However, for large-scale data, multi-sample binning across all samples becomes a resource-intensive process. We adapt single-sample binning for metaFun. The effectiveness and credibility of multi-sample are going to be investigated for further pipeline integration.

MetaBAT2 [57] has consistently shown competent performance in binning benchmark results, even in studies that introduce newer methods [58, 59]. MetaBAT2 is renowned for resource efficiency and speed. SemiBin2 utilizes either pre-trained models based on reference genomes or applies self-supervised learning that uses contigs to learn feature embeddings, and it showed robust performance.

Multiple binning methods can be applied simultaneously, and resulting MAGs can be combined and improved in quality through the bin refinement process. Representative bin refinement methods are DAS Tool [60], Binning_refiner [61], MetaWRAP [62], and BASALT [63]. DAS Tool performed best against MetaWRAP and Binning_refiner in a previous benchmark study[64]. BASALT utilized four modules to increase the number and quality of MAGs in a sample and harvested the most MAGs in number. But the total execution time of BASALT takes about four times that of DAS Tool. We selected MetaBAT2 and SemiBin2 for the binning method, and DAS Tool for bin refinement, and we evaluated the performances of binning/refining combinations to find out the optimal process in this study.

**Supplementary Note 1F. Selection of components for BIN_ASSESSMENT module**

MAG quality should be carefully examined before any downstream analysis, including comparative genomic analysis and MAG submissions to public repositories. Contaminated sequences reside in deposited genomes of public database [65], and MAGs are suspected to be a new source of contamination [66]. Mags are prone to contain contaminated information [67] and using incomplete MAGs can lead to incomplete functional inference [68]. Proper quality processing is critical when performing genomic analysis using MAGs. Quality assessment methods generally fall into several approaches: single-copy gene evaluation, database-free methods using genomic features, GC and taxonomy-specific *k*-mer analysis, and genome-wide matching [69]. For optimal results, the integration of multiple tools from these categories is recommended. Reporting the completeness and contamination scores of a MAG is a minimum requirement in the Minimum Information about a Metagenome-Assembled Genome (MIMAG) by the Genomic Standards Consortium [70]. CheckM2 [71] estimates completeness and contamination based on machine learning-based models constructed using RefSeq [72] genomes and KEGG [73] annotation results. GUNC [74] detects chimeric sequences and contamination using lineage information of contigs. Because CheckM2 and GUNC are recognized as standard programs for the assessment of MAG quality, we decided to utilize them for our pipeline for genome quality assessment.

**Supplementary Note 1G. Selection of components for COMPARATIVE_ANNOTATION module**

Comparative genomic analysis is a crucial process for understanding species functional capacities and ecological roles. A pangenome comprises core genes that are shared by most genomes and dispensable, strain-specific genes [75]. Because genomic variation in pangenomes significantly differs across species, multiple mechanisms have been proposed to explain microbial pangenomes. Conventional explanations on pangenomes, the conventional open and closed pangenomes, may not be sufficient to understand the genetic entity of species [76] and the openness of pangenomes could be temporarily related to sequenced genome set size [77]. In a recent experiment, the essentiality of genes in a species is not static and could be strain-dependent even in core genes [78]. Following previous perspectives and research results, we propose that species pangenomes should be analyzed with their associated genome datasets to understand their roles in their environment. In this module, we aimed to incorporate a pangenome analysis module for characterizing species pangenome with MAGs.

Compared to isolate genomes, MAGs tend to be more fragmented, incomplete, and contaminated. This leads to core gene loss and perturbs functional and phylogenetic analysis results. Consequently, parameters for comparative genomic analysis should be adequately managed when MAGs are included [79]. Several methods have been proposed to handle fragmented assemblies. GenAPI predicts gene presence and absence by lowering alignment coverage and increasing the identity threshold [80]. mOTUpan infers core genes by robust Bayesian method considering genome completeness [81]. PanDelos-frags selects genetically similar reference genome, integrates input contigs utilizing aligned regions, and infers gene presence status [82]. However, genetic content within a species is highly variable [67] and a genuine degree of genetic difference of analyzed genome to a reference genome is hard to identify. We decided to generate pangenome information using user-provided genomes without any inference on gene presence status or automated reference genome selection processes. Users can compose their genome dataset based on research objectives.

Sequence-clustering strategies include orthology-based, synteny-based, homology-based, and reference-based methods. The choice of method influences core gene, pangenome, and genetic diversity inference. There was no one-size-fits-all tool that excels in all tested metrics using low-quality genomes [83]. The homology-based method offers computational efficiency and robustness for pangenome analysis using a dataset containing low-quality genomes but shows limitations in paralog detection. We decided to utilize the homology-based clustering method for our pipeline. We decided to integrate PPanGGOLiN [84] into our pipeline, which utilizes the homology-based clustering method MMseqs2 enabling scalability. It is important to note that MAG is a consensus assembled genome from metagenomic reads, and this feature should be considered in downstream analyses [85]. Additional downstream analysis could be conducted with the generated pangenome, such as the addition of high-quality reference genomes to the pangenome and read mapping to it as a reference [86], although the read-mapping process is not included in our current pipeline. We decided to test the effect of gene family clustering thresholds and varying qualities of genome datasets on comparative genomic analyses.

For functional annotation of pangenome, we selected several widely used databases, which are evolutionary genealogy of genes: Non-supervised Orthologous Groups (eggNOG) [87], HMM database of KEGG Orthologs (KOfam) [88], Comprehensive Antibiotic Resistance Database (CARD) [89], Virulence Factor Database (VFDB) [90], and Carbohydrate-Active Enzymes (CAZy) [91]. eggNOG-mapper [92] identifies homologous genes and transfers annotated functions from the ortholog gene to the queried gene. KOfam is comprised of profile hidden Markov models built based on KEGG Orthologs, and HMMER [93] is utilized for profile searches. CARD includes antibiotic resistance genes and has a specialized ontology, Antibiotic Resistance Ontology (ARO), with Resistance Gene Identifier (RGI) to identify homologous genes using CARD. VFDB is a specialized database for virulence factors and homology search is performed by using DIAMOND [94]. dbCAN [95] harbors Carbohydrate active enzymes (CAZymes), HMMER, and DIAMOND are used for homology search. All utilized databases are carefully maintained, curated, and continuously updated by expert researchers. These databases are highly credible and accurate. eggNOG 5.0, KOfam built with KEGG release 112.0, dbCAN HMMdb V13, VFDB 2023, and CARD version 4.0.0 were utilized as functional databases in metaFun.

**Supplementary Note 1H. Selection of components for INTERACTIVE_TAXONOMY module**

The compositionality and zero-inflated characteristics of taxonomic composition data must be properly addressed to avoid spurious interpretations [96]. Data transformation affects downstream analysis, feature selection, and differential abundance analysis (DAA). No single transformation method guarantees optimal performance in all cases [97, 98]. Hundreds of R packages were developed for microbiome data analysis, and the visualization processes needed to be enhanced for analysis [99]. We decided to design an interactive module that supports diversity analysis and DAA of taxonomic composition with data transformation support. This module will enable exploratory data analysis of metagenomic data minimizing code-based execution.

Taxonomy profiling results with associated metadata are automatically packaged into phyloseq object [100], a standard object for microbiome analysis, in the WMS_TAXONOMY module. We selected microeco [101] as our data handling package. For diversity analysis, total sum scaling would preserve the original community structure among metagenomic datasets, but adequate data transformation is necessary for DAA to account for heteroskedasticity. [102, 103]. We support centered log-ratio transformation, log transformation, and default relative abundance-based analysis in this interactive module. Numerous DAA methods have been developed and benchmarked several times. Linear-based models have demonstrated robust performance in a previous study [104]. Conventional approaches such as LEfSe [105] or edgeR [106], which is originally developed for RNA-seq data, can produce many false positives without additional processing [107, 108]. A recent benchmark identified that proper control for confounding factors is an inevitable process for accurate analysis, and that traditional linear models and non-parametric statistical methods showed robust performance [109]. Consequently, we decided to utilize MaAsLin2 [110], which can control confounding factors and utilize linear models in interactive module.

**Supplementary Note 1I. Selection of components for WMS_STRAIN and INTERACTIVE_STRAIN modules**

Strain-level resolution is increasingly recognized as standard step for comprehensive metagenomic analysis, as species-level taxonomic profiling alone cannot capture the full extent of microbial functional and ecological diversity. Recent studies have demonstrated that intra-species genetic variations are associated with host phenotypes and ecological dynamics. For instance, bacterial single nucleotide variants (SNVs) within gut microbiome species have been significantly associated with host BMI, revealing phenotypic associations undetectable through relative abundance analysis alone [111]. Furthermore, strain richness varies considerably across species and directly affects engraftment outcomes in fecal microbiota transplantation [112]. Evolutionary modifications within species including SNV accumulation and gene loss/gain events, have been statistically linked to community compositional changes [113], supporting the "eco-evolutionary feedback" hypothesis in human gut ecosystems. These findings collectively establish that strain-level analysis is not merely a refinement but a prerequisite for accurate functional inference and ecological interpretation in metagenomics.

For strain-level profiling, we selected inStrain [114] as the primary tool for the WMS_STRAIN module. inStrain demonstrated exceptional accuracy in detecting major population variants enabling robust SNV calling even in complex metagenomic samples with moderate sequencing depth. The gene-level output structure of inStrain, including per-gene nucleotide diversity (π), SNV density, and ratio of nonsynonymous to synonymous polymorphisms (pN/pS), facilitates direct biological interpretation and downstream association analyses with metadata variables. Also, inStrain seamlessly integrates with species level representative reference genomes of GTDB, allowing coherent linkage between strain-level polymorphism data and species-level taxonomic frameworks established in the WMS_TAXONOMY module.

The interactive module, INTERACTIVE_STRAIN, provides rapid comprehensive population microdiversity of genomes and genes. For diversity analysis, π, SNV density, and ratio of pN/pS are computed at genome-wide and gene-specific levels, enabling detection of selective pressures. Coverage and breadth metrics are calculated to assess genome representation quality, with user-adjustable thresholds for filtering low-confidence calls. SNV density provides a normalized measure of genetic variation that facilitates cross species comparisons. For strain sharing analysis, population ANI (popANI) and consensus ANI (conANI) values are utilized to identify shared strains across samples, with configurable ANI thresholds. Statistical association test is implemented to identify relationships between strain-level metrics and categorical or numerical metadata variables. All strain-level analyses are implemented in an R Shiny interface, enabling interactive exploration, and customizable visualization with adjustable quality thresholds.

**Supplementary Note 1J. Selection of components for INTERACTIVE_NETWORK module**

DAA finds microbes that changes between conditions, but it overlooks emergent properties from their interconnections [115]. Co-occurrence network analysis addresses this limitation by revealing how microbial associations, including cooperative and competitive interactions, change in response to host phenotypes or environmental perturbations [116]. Network topology metrics such as modularity and connectivity have been statistically linked to ecosystem stability and functional redundancy [117], underscoring the necessity of incorporating network-level perspectives for comprehensive microbiome interpretation.

For network inference, we integrated two methods. FastSpar [118] was selected as the primary correlation-based approach, implementing a parallelized C++ version of the SparCC algorithm that explicitly addresses compositional bias inherent in relative abundance data while reducing computational time by two to three orders of magnitude compared to the original implementation. FlashWeave [119] was incorporated as a conditional independence-based method that distinguishes direct from indirect associations through probabilistic graphical model inference, substantially reducing false positive edges arising from transitive correlations.

INTERACTIVE_NETWORK module computes comprehensive network characterization metrics including node count, edge count, density, average degree, clustering coefficient, and modularity. For keystone taxon identification, we implemented the Zi-Pi classification framework, which categorizes nodes into peripheral specialists, module hubs, connectors, and network hubs based on within-module connectivity (Zi) and participation coefficient (Pi) [120]. The Integrated Value of Influence (IVI) algorithm [121] provides unbiased identification of influential nodes by combining six centrality measures, degree, Cluster Rank, neighborhood connectivity, local H-index, betweenness, and collective influence, capturing local, semi-local, and global topological dimensions simultaneously. Network robustness is evaluated through systematic node removal simulations using targeted attack strategies and random removal. The module tracks largest connected component size across removal fractions, computing area under the curve (AUC) and R50 metrics as quantitative stability indicators.

**Supplementary Note 1K. Selection of components for INTERACTIVE_COMPARATIVE module**

This module enables a rapid search of functional annotation results in a species pangenome and quick association analyses to identify relationships between genes and categorical or numerical phenotypes. Advances in genomic sequencing have greatly aided the characterization of antibiotic resistance, virulence factors, and genes associated with host adaptation or disease status [122, 123]. Although more extensive downstream analysis that integrates pangenome with metagenomic reads falls outside the scope of our current pipeline version, gene-, *k*-mer-based approaches and phylogenetic analysis could be harnessed for genome-wide association studies [124]. We decided to utilize gene-level data for association analysis and functional searches. pyseer [125] can stratify population structure using ANI distance and leverage multiple algorithms to identify associations on numerical metadata, benefiting from sustained tool development. Fisher's exact test is utilized for categorical metadata association. For exploratory and interactive analyses, this module performs dimension reduction via PCoA based on gene family presence, gene family tree inference, and interactive functional space search with ComplexHeatmap [126]. All functionalities are implemented in R shiny, enabling seamless integration with data generation by metaFun.

**Supplementary Note 2: Supplementary methods**

Supplementary Note 2A. Simulation of metagenomic reads using CAMI profiles

Fifteen microbial taxonomic abundance profiles with five samples from each of the three environments (human gut, rhizosphere, and marine) from the CAMI challenge were retrieved. All reference genomes used for metagenome simulation were downloaded from the CAMI repositories (Table 1) and were taxonomically assigned based on the Genome Taxonomy Database (GTDB) r220 using GTDB-Tk v2.4.0 [127]. NCBI TaxIds originally assigned to reference genomes were converted to GTDB TaxIds using gtdb-taxdump v0.5.0 (<https://github.com/shenwei356/gtdb-taxdump>) and TaxonKit v0.16.0 [128]. Metagenomic samples were simulated with a size of 5 GB using CAMISIM v1.3 [129] and read simulator ART v2.3.6 [130] following taxonomic retrieved profiles. All the taxonomic profiles and modified taxonomy information are deposited at <https://osf.io/xg9dj>.

Supplementary Note 2B. Performance evaluation of *de novo* assembly and binning/refining methods

Two *de novo* assemblers, which demonstrated decent performance in previous benchmark results on metagenomic data, were selected for performance evaluation (Supplementary Note 1E). MEGAHIT v1.2.9 with default option and meta-large of preset option, and metaSPAdes v3.15.5 with default option were used. Performance was evaluated using MetaQUAST v5.2.0 [131] with parameter '--unique-mapping –reuse-combined-alignments' to ensure accurate alignment. Performances of *de novo* assembly methods were evaluated with the six metrics of MetaQUAST: number of contigs, total length of assembled contigs, N50 value, total length of contigs containing misassembled region, fraction of aligned base pairs to reference genomes, and fraction of misassembled base pairs. All performance metrics of *de novo* assembly methods are available in Table S2.

For the binning process, MetaBAT2 v2.15, SemiBin2 v2.1.0, and DAS Tool v1.1.7 were selected (Supplementary Note 1E). MetaBAT2 was executed with the default options, and SemeBin2 was executed with two binning mode configurations: self-supervised learning mode ('*--self-supervised*') and prebuilt model-based mode ('*--environment soil, human_gut, ocean*'). DAS Tool was applied with '*--score_threshold=0*' option to combine and refine bins of MetaBAT2 and SemiBin2. Three binning and two binning with refinement methods were utilized to generate MAGs. Simulated metagenomic reads and GSAs generated by CAMISIM were utilized for binning and generated bins were assessed by accuracy metrics of AMBER v2.0.4 [132]. All binning/refining combinations were performed with a single-sample binning approach (Supplementary Note 1E). For the performance evaluation of binning/refining combinations, $Completeness(\%)$, and $Purity(\%)$ score of each genome, and $Accuracy\left( \% \right)$, adjusted Rand index (ARI) and F1 score ($F1_{AMBER}$) (t4) were calculated using AMBER. For every predicted genome bin, $Completeness(\%)$ and $Purity(\%)$ scores were calculated. Both scores range from 0 to 100. For bin $x$, False negatives ($FN_{x}$) represent the number of base pairs wrongly classified and true positives ($TP_{x}$) represent the number of base pairs that overlap with the mapped genome.

$Completeness(\%)=\frac{TP_{x}}{TP_{x}+FN_{x}} * 100$ *(1)*

The $Purity (\%)$ of bin represents the ratio of base pairs correctly assigned base pairs ($TP_{x}$) among the sum of number of base pairs belonging to other genomes ($FP_{x}$) and $TP_{x}$ in each bin.

$Purity(\%)=\frac{TP_{x}}{TP_{x}+FP_{x}} * 100$ *(2)*

The $Accuracy\left( \% \right)$ represents accuracy of base pairs (%) over each metagenomic sample. $U$ is the number of unassigned base pairs and $X$ is the generated bin set. $TP_{x}$ and $FP_{x}$ denote true positives and false positives for predicted bin $x$.

$Accuracy\left( \% \right)=\frac{\Sigma_{x \in X}TPx}{U + \Sigma_{x \in X}\left( TPx + FPx \right)} * 100$ *(3)*

The ARI defined in AMBER, represents clustering resolution of binned base pairs to the gold standard mapping file correctly. The F1 score ($F1_{AMBER}$) represents the accuracy of binned base pairs in a sample by calculating correctly assigned base pairs to bins. $F1_{AMBER}$ is the harmonic mean of recall and precision in a sample: $TP$ (True positive contig), $FP$ (False positive contig), and $FN$ (False negative contigs) ARI and $F1_{AMBER}$ score range from 0 to 1.

$F1_{AMBER}=2 * \frac{\frac{TP}{TP+FP}*\frac{TP}{TP+FN}}{\frac{TP}{TP+FP}+\frac{TP}{TP+FN}}$ *(4)*

Generation of MAG datasets for evaluating comparative genomic analysis
All complete NCBI assembly-level genomes from species in GTDB r214 that have at least 50 complete NCBI assembly-level genomes were downloaded using NCBI datasets cli v15.25.0 [133]. A total of 67 species satisfied the above condition, leading to the retrieval of 14,875 complete genomes. In each species, the top 50 genomes were selected based on the ${QS}_{50}$ + ln(N50) score, which integrates the completeness and contamination scores assessed by CheckM2 and N50 value of each genome. N50 is calculated by first ordering all contigs by length, then adding up contig lengths until reaching 50% of the total genome size. The last added contig length is N50 contig value at which 50% threshold is reached. This resulted in a total of 3,350 genomes.

${QS}_{50}= completeness - 5 \times contamination$ *(5)*

To generate genomes that emulate MAG features (i.e., fragmented and incomplete genomes), 3,350 genomes were randomly fragmented to make a "Fragmented" genome dataset. Second dataset was generated with random fragmentation and the removal of 5% from each contig end, totaling 10% deletion. Fragmentation followed a normal distribution with mean contig length at 1% of genome length and standard deviation at 25% of mean. Genome quality of three datasets was assessed using CheckM2. We refer to these three genome datasets as "Complete", "Fragmented”, and "Fragmented Incomplete" datasets in the main text.

Supplementary Note 2C. Evaluation of the impact of parameter selection on pangenome-based functional annotation and comparative genomic analysis

Coding sequences (CDSs) of all genomes in the three genome datasets were predicted using Prokka v1.14.6 [134]. PPanGGOLiN v2.0.5 was utilized for comparative genomic analysis. All pangenome and comparative genomic analyses were conducted at the species level. To assess the effect of gene family clustering threshold on analysis results, two thresholds were applied: 80% identity 80% coverage, and 90% identity 50% coverage. The number of gene families in a species was counted (Table S4). KofamScan v1.3.0 was used to annotate the genome datasets based on the KOfam database (HMM database of KEGG Orthologs) version of 27Nov2023. The pangenome-based functional annotation approach uses the reference gene family annotation of a species to assign functional information to individual genomes via the gene family count matrix. The impact of parameter choice on the accuracy of pangenome-based functional annotation was assessed. 3,350 complete genomes were annotated individually. For each species, the functional profiles of 50 genomes were merged into a species reference functional profile, and the profiles of pangenome-based functional annotation from three genome datasets were compared to 67 species reference functional profiles. The result of pangenome-based KO annotation ($KO_{pan,i}$) was compared to individual reference KO annotation result ($KO_{ref,i}$) for species $i$. The sum of the differences of KO count in each pair ($\sum_{i=1}^{50} \left| KO_{ref,i}-KO_{pan,i} \right|$) was divided by the sum of complete genome $CDS$s (coding sequences) ($\sum_{i=1}^{50} CDS_{ref,i}$). We refer to $D_{KO}$ as normalized Manhattan distance in the text.

$D_{KO}=\frac{\sum_{i=1}^{50} \left| KO_{ref,i}-KO_{pan,i} \right|}{\sum_{i=1}^{50} CDS_{ref,i}}$ *(6)*

The presence-absence of pangenome-based KOs was compared to derive the F1 score ($F1_{KO}$). $TP_{KO}$ represents the number of correctly assigned KOs via pangenome-based annotation. $FP_{KO}$ represents the number of absent KOs incorrectly annotated as present in the pangenome-based annotation. ${FN}_{KO}$ represents the number of present KOs incorrectly annotated as absent in pangenome-based annotation (Table S5).

$F1_{KO} =\frac{2 \cdot{TP}_{KO}}{2 \cdot{TP}_{KO}+ {FP}_{KO}+ {FN}_{KO}}$ *(7)*

Supplementary Note 2D. Evaluation of the impact of parameter selection on genome fluidity and core genome analysis

The genome fluidity [135] value was calculated with PPanGGOLiN using gene family information in a species. We assessed the genome fluidity pattern with varying clustering thresholds of gene families and three genome datasets. Due to computing resource limitations, ten out of 67 species were selected at regular intervals of genome fluidity from the lowest to the highest for accuracy evaluation of core genome tree inference. The number of core gene families and phylogenetic tree structure were compared within each species to identify the impact of genome quality, gene family clustering threshold, and core gene threshold on analysis results (Table S6). Concatenated core nucleotide gene alignments were generated using MAFFT [136] with three core gene thresholds (90%, 95%, and 99%), and single-nucleotide polymorphism (SNP) information was extracted using snp-sites [137]. All phylogenetic trees were inferred using FastTree v2.1.11 [138] with the Jukes-Cantor model and option '*-fastest -nt*'. The topology of any pair of trees in a species was compared to assess the impact of gene family clustering thresholds, genome quality and core gene threshold on phylogenetic tree inference accuracy. The patristic distance matrix in a tree was calculated using the cophenetic function of ape v5.7.1 [139]. The Mantel test (Spearman correlation, 999 permutations) was conducted to compare a pair of distance matrices using vegan v2.6.4 [140].

Supplementary Note 2E. Construction of taxonomy-profiling databases with GTDB r220 and detected genomes, and performance evaluation of varying parameters

For taxonomic profiling of metagenomes, a new Kraken2, Bracken, and sylph databases were built with 113,104 species representative genomes of GTDB r220. The TaxIDs of 107,235 bacterial species and 5,869 archaeal species were created with the GTDB r220 file using gtdb-taxdump v0.5.0. The Kraken2 database was built using Kraken2 v2.1.3. Bracken2 databases were built with read lengths of 100, 150, 200, 250, and 300 bp using Bracken version 2.9. Gold taxonomic profiles, which are taxonomic abundance profiles, were generated with GTDB r220 taxonomy information using CAMISIM. Sequence abundance gold profiles were generated by calculating the raw sequence proportions in simulated metagenomes. We evaluated the effects of varying confidence values (0, 0.1, 0.25, 0.5) of Kraken2 and relative abundance filtration thresholds (0%, 0.01%, 0.001%, 0.0001%) of Bracken on the accuracy of taxonomic profiling using OPAL v1.0.12 [141]. L1 norm, Bray-Curtis dissimilarity, completeness, purity, and F1 score were assessed to evaluate the performance of taxonomic profiling at each taxonomic rank unit from Kingdom to Species. sylph parameters (compression rate, minimum number of *k*-mer, and unknown estimation status) varied when evaluating performance using the three metrics (Table S7). L1 norm represents the total error between true and predicted relative abundance at any taxonomic rank. Bray-Curtis dissimilarity is obtained by dividing the sum of absolute pairwise differences in abundance of each taxon by the sum of abundances across all taxa. Purity represents the ratio of correctly predicted taxa in the profiled sample. Completeness represents the ratio of correctly predicted taxa to the total ground truth taxa. F1 score is the harmonic average of completeness and purity. The ranking system of OPAL was utilized to select accurate taxonomic profiling parameters of the profilers. The effect of taxonomic coverage of the databases was assessed using sylph. Environment-specific genome databases were compared with the GTDB r220 genome-based database constructed using sylph with c200 parameter using evaluation metrics.

**References**

1. Quince C, Walker AW, Simpson JT et al. Shotgun metagenomics, from sampling to analysis, Nature Biotechnology 2017;35:833-844.

2. Yang C, Chowdhury D, Zhang Z et al. A review of computational tools for generating metagenome-assembled genomes from metagenomic sequencing data, Computational and Structural Biotechnology Journal 2021;19:6301-6314.

3. Gardner PP, Paterson JM, McGimpsey S et al. Sustained software development, not number of citations or journal choice, is indicative of accurate bioinformatic software, Genome biology 2022;23:56.

4. Knight R, Vrbanac A, Taylor BC et al. Best practices for analysing microbiomes, Nature Reviews Microbiology 2018;16:410-422.

5. Saheb Kashaf S, Almeida A, Segre JA et al. Recovering prokaryotic genomes from host-associated, short-read shotgun metagenomic sequencing data, Nature Protocols 2021;16:2520-2541.

6. Chivian D, Jungbluth SP, Dehal PS et al. Metagenome-assembled genome extraction and analysis from microbiomes using KBase, Nature Protocols 2023;18:208-238.

7. Brooks TG, Lahens NF, Mrčela A et al. Challenges and best practices in omics benchmarking, Nature Reviews Genetics 2024;25:326-339.

8. Stoler N, Nekrutenko A. Sequencing error profiles of Illumina sequencing instruments, NAR Genomics and Bioinformatics 2021;3:lqab019.

9. Del Fabbro C, Scalabrin S, Morgante M et al. An Extensive Evaluation of Read Trimming Effects on Illumina NGS Data Analysis, PLoS One 2013;8:e85024.

10. Pereira-Marques J, Hout A, Ferreira RM et al. Impact of Host DNA and Sequencing Depth on the Taxonomic Resolution of Whole Metagenome Sequencing for Microbiome Analysis, Frontiers in Microbiology 2019;10:1277.

11. Chen S, Zhou Y, Chen Y et al. fastp: an ultra-fast all-in-one FASTQ preprocessor, Bioinformatics 2018;34:i884-i890.

12. Martin M. Cutadapt removes adapter sequences from high-throughput sequencing reads, EMBnet. journal 2011;17:10-12.

13. Bolger AM, Lohse M, Usadel B. Trimmomatic: a flexible trimmer for Illumina sequence data, Bioinformatics 2014;30:2114-2120.

14. He B, Zhu R, Yang H et al. Assessing the Impact of Data Preprocessing on Analyzing Next Generation Sequencing Data, Frontiers in Bioengineering and Biotechnology 2020;8:817.

15. Langmead B, Salzberg SL. Fast gapped-read alignment with Bowtie 2, Nature Methods 2012;9:357-359.

16. Li H, Durbin R. Fast and accurate short read alignment with Burrows–Wheeler transform, Bioinformatics 2009;25:1754-1760.

17. Lee H, Lee K-W, Lee T et al. Performance evaluation method for read mapping tool in clinical panel sequencing, Genes & Genomics 2018;40:189-197.

18. Rumbavicius I, Rounge TB, Rognes T. HoCoRT: host contamination removal tool, BMC Bioinformatics 2023;24:371.

19. Ewels P, Magnusson M, Lundin S et al. MultiQC: summarize analysis results for multiple tools and samples in a single report, Bioinformatics 2016;32:3047-3048.

20. Sun Z, Huang S, Zhang M et al. Challenges in benchmarking metagenomic profilers, Nature Methods 2021;18:618-626.

21. Blanco-Míguez A, Beghini F, Cumbo F et al. Extending and improving metagenomic taxonomic profiling with uncharacterized species using MetaPhlAn 4, Nature Biotechnology 2023;41:1633-1644.

22. Ruscheweyh H-J, Milanese A, Paoli L et al. Cultivation-independent genomes greatly expand taxonomic-profiling capabilities of mOTUs across various environments, Microbiome 2022;10:212.

23. Wood DE, Lu J, Langmead B. Improved metagenomic analysis with Kraken 2, Genome biology 2019;20:257.

24. Lu J, Breitwieser FP, Thielen P et al. Bracken: estimating species abundance in metagenomics data, Peerj Computer Science 2017;3:e104.

25. Menzel P, Ng KL, Krogh A. Fast and sensitive taxonomic classification for metagenomics with Kaiju, Nature communications 2016;7:11257.

26. Steinegger M, Söding J. MMseqs2 enables sensitive protein sequence searching for the analysis of massive data sets, Nature Biotechnology 2017;35:1026-1028.

27. Ye SH, Siddle KJ, Park DJ et al. Benchmarking Metagenomics Tools for Taxonomic Classification, Cell 2019;178:779-794.

28. Meyer F, Fritz A, Deng Z-L et al. Critical Assessment of Metagenome Interpretation: the second round of challenges, Nature Methods 2022;19:429-440.

29. LaPierre N, Alser M, Eskin E et al. Metalign: efficient alignment-based metagenomic profiling via containment min hash, Genome biology 2020;21:242.

30. Valencia EM, Maki KA, Dootz JN et al. Mock community taxonomic classification performance of publicly available shotgun metagenomics pipelines, Scientific Data 2024;11:81.

31. Puller V, Plaza Oñate F, Prifti E et al. Impact of simulation and reference catalogues on the evaluation of taxonomic profiling pipelines, Microbial Genomics 2025;11:001330.

32. Bradford LM, Carrillo C, Wong A. Managing false positives during detection of pathogen sequences in shotgun metagenomics datasets, BMC Bioinformatics 2024;25:372.

33. Wright RJ, Comeau AM, Langille MGI. From defaults to databases: parameter and database choice dramatically impact the performance of metagenomic taxonomic classification tools, Microbial Genomics 2023;9:000949.

34. Edwin NR, Fitzpatrick AH, Brennan F et al. An in-depth evaluation of metagenomic classifiers for soil microbiomes, Environmental Microbiome 2024;19:19.

35. Shaw J, Yu YW. Rapid species-level metagenome profiling and containment estimation with sylph, Nature Biotechnology 2024:1-12.

36. Parks DH, Chuvochina M, Rinke C et al. GTDB: an ongoing census of bacterial and archaeal diversity through a phylogenetically consistent, rank normalized and complete genome-based taxonomy, Nucleic acids research 2021;50:D785-D794.

37. Bosilj M, Suljič A, Zakotnik S et al. MetaAll: integrative bioinformatics workflow for analysing clinical metagenomic data, Briefings in Bioinformatics 2024;25:bbae597.

38. Zafeiropoulos H, Beracochea M, Ninidakis S et al. metaGOflow: a workflow for the analysis of marine Genomic Observatories shotgun metagenomics data, Gigascience 2023;12:giad078.

39. Commichaux S, Shah N, Ghurye J et al. A critical assessment of gene catalogs for metagenomic analysis, Bioinformatics 2021;37:2848-2857.

40. Beghini F, McIver LJ, Blanco-Míguez A et al. Integrating taxonomic, functional, and strain-level profiling of diverse microbial communities with bioBakery 3, Elife 2021;10:e65088.

41. Nayfach S, Shi ZJ, Seshadri R et al. New insights from uncultivated genomes of the global human gut microbiome, Nature 2019;568:505-510.

42. Delgado LF, Andersson AF. Evaluating metagenomic assembly approaches for biome-specific gene catalogues, Microbiome 2022;10:72.

43. Vosloo S, Huo L, Anderson CL et al. Evaluating de novo assembly and binning strategies for time series drinking water metagenomes, Microbiology Spectrum 2021;9:e01434-01421.

44. Mendes CI, Vila-Cerqueira P, Motro Y et al. LMAS: evaluating metagenomic short de novo assembly methods through defined communities, Gigascience 2022;12:giac122.

45. Zhang Z, Yang C, Veldsman WP et al. Benchmarking genome assembly methods on metagenomic sequencing data, Briefings in Bioinformatics 2023;24:1-17.

46. Li D, Luo R, Liu C-M et al. MEGAHIT v1.0: A fast and scalable metagenome assembler driven by advanced methodologies and community practices, Methods 2016;102:3-11.

47. Nurk S, Meleshko D, Korobeynikov A et al. metaSPAdes: a new versatile metagenomic assembler, Genome research 2017;27:824-834.

48. Wang Z, Wang Y, Fuhrman JA et al. Assessment of metagenomic assemblers based on hybrid reads of real and simulated metagenomic sequences, Briefings in Bioinformatics 2019;21:777-790.

49. Jochheim A, Jochheim FA, Kolodyazhnaya A et al. Strain-resolved de-novo metagenomic assembly of viral genomes and microbial 16S rRNAs, Microbiome 2024;12:187.

50. Mise K, Iwasaki W. Unexpected absence of ribosomal protein genes from metagenome-assembled genomes, ISME Communications 2022;2:118.

51. Kerkvliet JJ, Bossers A, Kers JG et al. Metagenomic assembly is the main bottleneck in the identification of mobile genetic elements, PeerJ 2024;12:e16695.

52. Abramova A, Karkman A, Bengtsson-Palme J. Metagenomic assemblies tend to break around antibiotic resistance genes, BMC Genomics 2024;25:959.

53. Mallawaarachchi V, Wickramarachchi A, Xue H et al. Solving genomic puzzles: computational methods for metagenomic binning, Briefings in Bioinformatics 2024;25:bbae372.

54. Nissen JN, Johansen J, Allesøe RL et al. Improved metagenome binning and assembly using deep variational autoencoders, Nature Biotechnology 2021;39:555-560.

55. Pan S, Zhao X-M, Coelho LP. SemiBin2: self-supervised contrastive learning leads to better MAGs for short- and long-read sequencing, Bioinformatics 2023;39:i21-i29.

56. Mattock J, Watson M. A comparison of single-coverage and multi-coverage metagenomic binning reveals extensive hidden contamination, Nature Methods 2023;20:1170-1173.

57. Kang DD, Li F, Kirton E et al. MetaBAT 2: an adaptive binning algorithm for robust and efficient genome reconstruction from metagenome assemblies, PeerJ 2019;7:e7359.

58. Wang Z, Huang P, You R et al. MetaBinner: a high-performance and stand-alone ensemble binning method to recover individual genomes from complex microbial communities, Genome biology 2023;24:1.

59. Wang Z, You R, Han H et al. Effective binning of metagenomic contigs using contrastive multi-view representation learning, Nature communications 2024;15:585.

60. Sieber CMK, Probst AJ, Sharrar A et al. Recovery of genomes from metagenomes via a dereplication, aggregation and scoring strategy, Nature Microbiology 2018;3:836-843.

61. Song W-Z, Thomas T. Binning_refiner: improving genome bins through the combination of different binning programs, Bioinformatics 2017;33:1873-1875.

62. Uritskiy GV, DiRuggiero J, Taylor J. MetaWRAP—a flexible pipeline for genome-resolved metagenomic data analysis, Microbiome 2018;6:158.

63. Qiu Z, Yuan L, Lian C-A et al. BASALT refines binning from metagenomic data and increases resolution of genome-resolved metagenomic analysis, Nature communications 2024;15:2179.

64. Yue Y, Huang H, Qi Z et al. Evaluating metagenomics tools for genome binning with real metagenomic datasets and CAMI datasets, BMC Bioinformatics 2020;21:334.

65. Steinegger M, Salzberg SL. Terminating contamination: large-scale search identifies more than 2,000,000 contaminated entries in GenBank, Genome biology 2020;21:115.

66. Astashyn A, Tvedte ES, Sweeney D et al. Rapid and sensitive detection of genome contamination at scale with FCS-GX, Genome biology 2024;25:60.

67. Meziti A, Rodriguez-R LM, Hatt JK et al. The reliability of Metagenome-Assembled Genomes (MAGs) in representing natural populations: Insights from comparing mags against isolate genomes derived from the same fecal sample, Applied and Environmental Microbiology 2021;87:e02593-02520.

68. Eisenhofer R, Odriozola I, Alberdi A. Impact of microbial genome completeness on metagenomic functional inference, ISME Communications 2023;3:12.

69. Cornet L, Baurain D. Contamination detection in genomic data: more is not enough, Genome biology 2022;23:60.

70. Bowers RM, Kyrpides NC, Stepanauskas R et al. Minimum information about a single amplified genome (MISAG) and a metagenome-assembled genome (MIMAG) of bacteria and archaea, Nature Biotechnology 2017;35:725-731.

71. Chklovski A, Parks DH, Woodcroft BJ et al. CheckM2: a rapid, scalable and accurate tool for assessing microbial genome quality using machine learning, Nature Methods 2023;20:1203-1212.

72. Haft DH, DiCuccio M, Badretdin A et al. RefSeq: an update on prokaryotic genome annotation and curation, Nucleic acids research 2017;46:D851-D860.

73. Kanehisa M, Goto S. KEGG: Kyoto Encyclopedia of Genes and Genomes, Nucleic acids research 2000;28:27-30.

74. Orakov A, Fullam A, Coelho LP et al. GUNC: detection of chimerism and contamination in prokaryotic genomes, Genome biology 2021;22:178.

75. Tettelin H, Masignani V, Cieslewicz MJ et al. Genome analysis of multiple pathogenic isolates of *Streptococcus agalactiae*: Implications for the microbial “pan-genome”, Proceedings of the National Academy of Sciences 2005;102:13950-13955.

76. Domingo-Sananes MR, McInerney JO. Mechanisms That Shape Microbial Pangenomes, Trends in Microbiology 2021;29:493-503.

77. Zhou Z, Charlesworth J, Achtman M. Accurate reconstruction of bacterial pan- and core genomes with PEPPAN, Genome research 2020;30:1667-1679.

78. Rosconi F, Rudmann E, Li J et al. A bacterial pan-genome makes gene essentiality strain-dependent and evolvable, Nature Microbiology 2022;7:1580-1592.

79. Li T, Yin Y. Critical assessment of pan-genomic analysis of metagenome-assembled genomes, Briefings in Bioinformatics 2022;23:1–12.

80. Gabrielaite M, Marvig RL. GenAPI: a tool for gene absence-presence identification in fragmented bacterial genome sequences, BMC Bioinformatics 2020;21:320.

81. Buck M, Mehrshad M, Bertilsson S. mOTUpan: a robust Bayesian approach to leverage metagenome-assembled genomes for core-genome estimation, NAR Genomics and Bioinformatics 2022;4:lqac060.

82. Bonnici V, Mengoni C, Mangoni M et al. PanDelos-frags: A methodology for discovering pangenomic content of incomplete microbial assemblies, Journal of Biomedical Informatics 2023;148:104552.

83. Manzano-Morales S, Liu Y, González-Bodí S et al. Comparison of gene clustering criteria reveals intrinsic uncertainty in pangenome analyses, Genome biology 2023;24:250.

84. Gautreau G, Bazin A, Gachet M et al. PPanGGOLiN: Depicting microbial diversity via a partitioned pangenome graph, PLoS computational biology 2020;16:e1007732.

85. Van Rossum T, Ferretti P, Maistrenko OM et al. Diversity within species: interpreting strains in microbiomes, Nature Reviews Microbiology 2020;18:491-506.

86. Matthews CA, Watson-Haigh NS, Burton RA et al. A gentle introduction to pangenomics, Briefings in Bioinformatics 2024;25:bbae588.

87. Huerta-Cepas J, Szklarczyk D, Heller D et al. eggNOG 5.0: a hierarchical, functionally and phylogenetically annotated orthology resource based on 5090 organisms and 2502 viruses, Nucleic acids research 2019;47:D309-D314.

88. Aramaki T, Blanc-Mathieu R, Endo H et al. KofamKOALA: KEGG Ortholog assignment based on profile HMM and adaptive score threshold, Bioinformatics 2019;36:2251-2252.

89. Alcock BP, Huynh W, Chalil R et al. CARD 2023: expanded curation, support for machine learning, and resistome prediction at the Comprehensive Antibiotic Resistance Database, Nucleic acids research 2022;51:D690-D699.

90. Zhou S, Liu B, Zheng D et al. VFDB 2025: an integrated resource for exploring anti-virulence compounds, Nucleic acids research 2025;53:D871-D877.

91. Drula E, Garron M-L, Dogan S et al. The carbohydrate-active enzyme database: functions and literature, Nucleic acids research 2021;50:D571-D577.

92. Cantalapiedra CP, Hernández-Plaza A, Letunic I et al. eggNOG-mapper v2: Functional Annotation, Orthology Assignments, and Domain Prediction at the Metagenomic Scale, Molecular Biology and Evolution 2021;38:5825-5829.

93. Eddy SR. Accelerated Profile HMM Searches, PLoS computational biology 2011;7:e1002195.

94. Buchfink B, Reuter K, Drost H-G. Sensitive protein alignments at tree-of-life scale using DIAMOND, Nature Methods 2021;18:366-368.

95. Zheng J, Ge Q, Yan Y et al. dbCAN3: automated carbohydrate-active enzyme and substrate annotation, Nucleic acids research 2023;51:W115-W121.

96. Gloor GB, Macklaim JM, Pawlowsky-Glahn V et al. Microbiome Datasets Are Compositional: And This Is Not Optional, Frontiers in Microbiology 2017;8:2224.

97. Yerke A, Fry Brumit D, Fodor AA. Proportion-based normalizations outperform compositional data transformations in machine learning applications, Microbiome 2024;12:45.

98. Wang B, Sun F, Luan Y. Comparison of the effectiveness of different normalization methods for metagenomic cross-study phenotype prediction under heterogeneity, Scientific Reports 2024;14:7024.

99. Wen T, Niu G, Chen T et al. The best practice for microbiome analysis using R, Protein & Cell 2023;14:713-725.

100. McMurdie PJ, Holmes S. phyloseq: An R Package for Reproducible Interactive Analysis and Graphics of Microbiome Census Data, PLoS One 2013;8:e61217.

101. Liu C, Cui Y, Li X et al. microeco: an R package for data mining in microbial community ecology, FEMS Microbiology Ecology 2020;97:fiaa255.

102. Chong J, Liu P, Zhou G et al. Using MicrobiomeAnalyst for comprehensive statistical, functional, and meta-analysis of microbiome data, Nature Protocols 2020;15:799-821.

103. McKnight DT, Huerlimann R, Bower DS et al. Methods for normalizing microbiome data: An ecological perspective, Methods in Ecology and Evolution 2019;10:389-400.

104. Yang L, Chen J. Benchmarking differential abundance analysis methods for correlated microbiome sequencing data, Briefings in Bioinformatics 2023;24:bbac607.

105. Segata N, Izard J, Waldron L et al. Metagenomic biomarker discovery and explanation, Genome biology 2011;12:R60.

106. Robinson MD, McCarthy DJ, Smyth GK. edgeR: a Bioconductor package for differential expression analysis of digital gene expression data, Bioinformatics 2009;26:139-140.

107. Yang L, Chen J. A comprehensive evaluation of microbial differential abundance analysis methods: current status and potential solutions, Microbiome 2022;10:130.

108. Nearing JT, Douglas GM, Hayes MG et al. Microbiome differential abundance methods produce different results across 38 datasets, Nature communications 2022;13:342.

109. Wirbel J, Essex M, Forslund SK et al. A realistic benchmark for differential abundance testing and confounder adjustment in human microbiome studies, Genome biology 2024;25:247.

110. Mallick H, Rahnavard A, McIver LJ et al. Multivariable association discovery in population-scale meta-omics studies, PLoS computational biology 2021;17:e1009442.

111. Zahavi L, Lavon A, Reicher L et al. Bacterial SNPs in the human gut microbiome associate with host BMI, Nat Med 2023;29:2785-2792.

112. Chen-Liaw A, Aggarwala V, Mogno I et al. Gut microbiota strain richness is species specific and affects engraftment (vol 637, pg 422, 2025), Nature 2025;638:E4-E4.

113. Madi N, Chen D, Wolff R et al. Community diversity is associated with intra-species genetic diversity and gene loss in the human gut microbiome, Elife 2023;12.

114. Olm MR, Crits-Christoph A, Bouma-Gregson K et al. inStrain profiles population microdiversity from metagenomic data and sensitively detects shared microbial strains, Nat Biotechnol 2021;39:727-736.

115. Kajihara KT, Hynson NA. Networks as tools for defining emergent properties of microbiomes and their stability, Microbiome 2024;12.

116. Faust K, Raes J. Microbial interactions: from networks to models, Nature Reviews Microbiology 2012;10:538-550.

117. Matchado MS, Lauber M, Reitmeier S et al. Network analysis methods for studying microbial communities: A mini review, Comput Struct Biotechnol J 2021;19:2687-2698.

118. Watts SC, Ritchie SC, Inouye M et al. FastSpar: rapid and scalable correlation estimation for compositional data, Bioinformatics 2019;35:1064-1066.

119. Tackmann J, Matias Rodrigues JF, von Mering C. Rapid Inference of Direct Interactions in Large-Scale Ecological Networks from Heterogeneous Microbial Sequencing Data, Cell Syst 2019;9:286-296 e288.

120. Guimerà R, Amaral LAN. Functional cartography of complex metabolic networks, Nature 2005;433:895-900.

121. Salavaty A, Ramialison M, Currie PD. Integrated Value of Influence: An Integrative Method for the Identification of the Most Influential Nodes within Networks, Patterns 2020;1:100052.

122. Power RA, Parkhill J, de Oliveira T. Microbial genome-wide association studies: lessons from human GWAS, Nature Reviews Genetics 2017;18:41-50.

123. Sheppard SK, Guttman DS, Fitzgerald JR. Population genomics of bacterial host adaptation, Nature Reviews Genetics 2018;19:549-565.

124. Allen JP, Snitkin E, Pincus NB et al. Forest and Trees: Exploring Bacterial Virulence with Genome-wide Association Studies and Machine Learning, Trends in Microbiology 2021;29:621-633.

125. Lees JA, Galardini M, Bentley SD et al. pyseer: a comprehensive tool for microbial pangenome-wide association studies, Bioinformatics 2018;34:4310-4312.

126. Gu Z. Complex heatmap visualization, Imeta 2022;1:e43.

127. Chaumeil P-A, Mussig AJ, Hugenholtz P et al. GTDB-Tk v2: memory friendly classification with the genome taxonomy database, Bioinformatics 2022;38:5315-5316.

128. Shen W, Ren H. TaxonKit: A practical and efficient NCBI taxonomy toolkit, Journal of Genetics and Genomics 2021;48:844-850.

129. Fritz A, Hofmann P, Majda S et al. CAMISIM: simulating metagenomes and microbial communities, Microbiome 2019;7:17.

130. Huang W, Li L, Myers JR et al. ART: a next-generation sequencing read simulator, Bioinformatics 2011;28:593-594.

131. Mikheenko A, Saveliev V, Gurevich A. MetaQUAST: evaluation of metagenome assemblies, Bioinformatics 2015;32:1088-1090.

132. Meyer F, Hofmann P, Belmann P et al. AMBER: Assessment of Metagenome BinnERs, Gigascience 2018;7:giy069.

133. Sayers EW, Bolton EE, Brister JR et al. Database resources of the national center for biotechnology information, Nucleic acids research 2021;50:D20-D26.

134. Seemann T. Prokka: rapid prokaryotic genome annotation, Bioinformatics 2014;30:2068-2069.

135. Kislyuk AO, Haegeman B, Bergman NH et al. Genomic fluidity: an integrative view of gene diversity within microbial populations, BMC Genomics 2011;12:32.

136. Katoh K, Misawa K, Kuma Ki et al. MAFFT: a novel method for rapid multiple sequence alignment based on fast Fourier transform, Nucleic acids research 2002;30:3059-3066.

137. Page AJ, Taylor B, Delaney AJ et al. SNP-sites: rapid efficient extraction of SNPs from multi-FASTA alignments, Microbial Genomics 2016;2.

138. Price MN, Dehal PS, Arkin AP. FastTree 2 – Approximately Maximum-Likelihood Trees for Large Alignments, PLoS One 2010;5:e9490.

139. Paradis E, Schliep K. ape 5.0: an environment for modern phylogenetics and evolutionary analyses in R, Bioinformatics 2018;35:526-528.

140. Dixon P. VEGAN, a package of R functions for community ecology, Journal of Vegetation Science 2003;14:927-930.

141. Meyer F, Bremges A, Belmann P et al. Assessing taxonomic metagenome profilers with OPAL, Genome biology 2019;20:51.

**Supplementary figures**


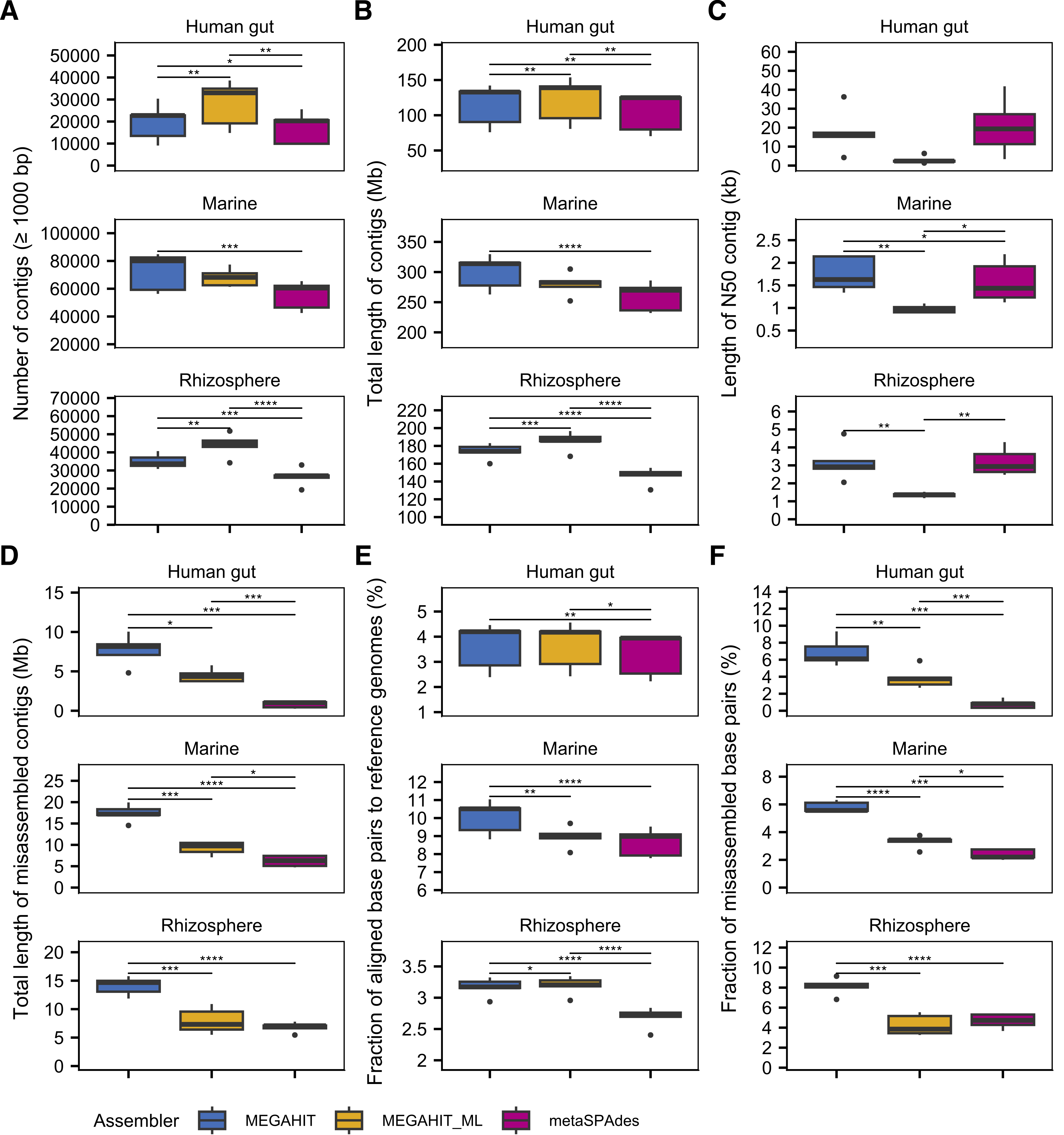


**Supplementary Figure 1. Performance evaluation of the assembly methods on the CAMI metagenomic dataset.** MEGAHIT with the default or meta-large option, or metaSPAdes with the default option was used for the *de novo* assembly of simulated metagenomes from three environments. **A**-**F** The quality of assembled contigs is represented with the box plots with several metrics: (**A**) number of contigs, (**B**) total length of assembled contigs, (**C**) N50 value, (**D**) total length of contigs containing misassembled region, (**E**) fraction of aligned base pairs to reference genomes, and (**F**) fraction of misassembled base pairs. MEGAHIT_ML represents assemblies generated with MEGAHIT with the meta-large option. (**P* < 0.05, ***P* < 0.01, ****P* < 0.001, *****P* < 0.0001; paired *t*-test)

**
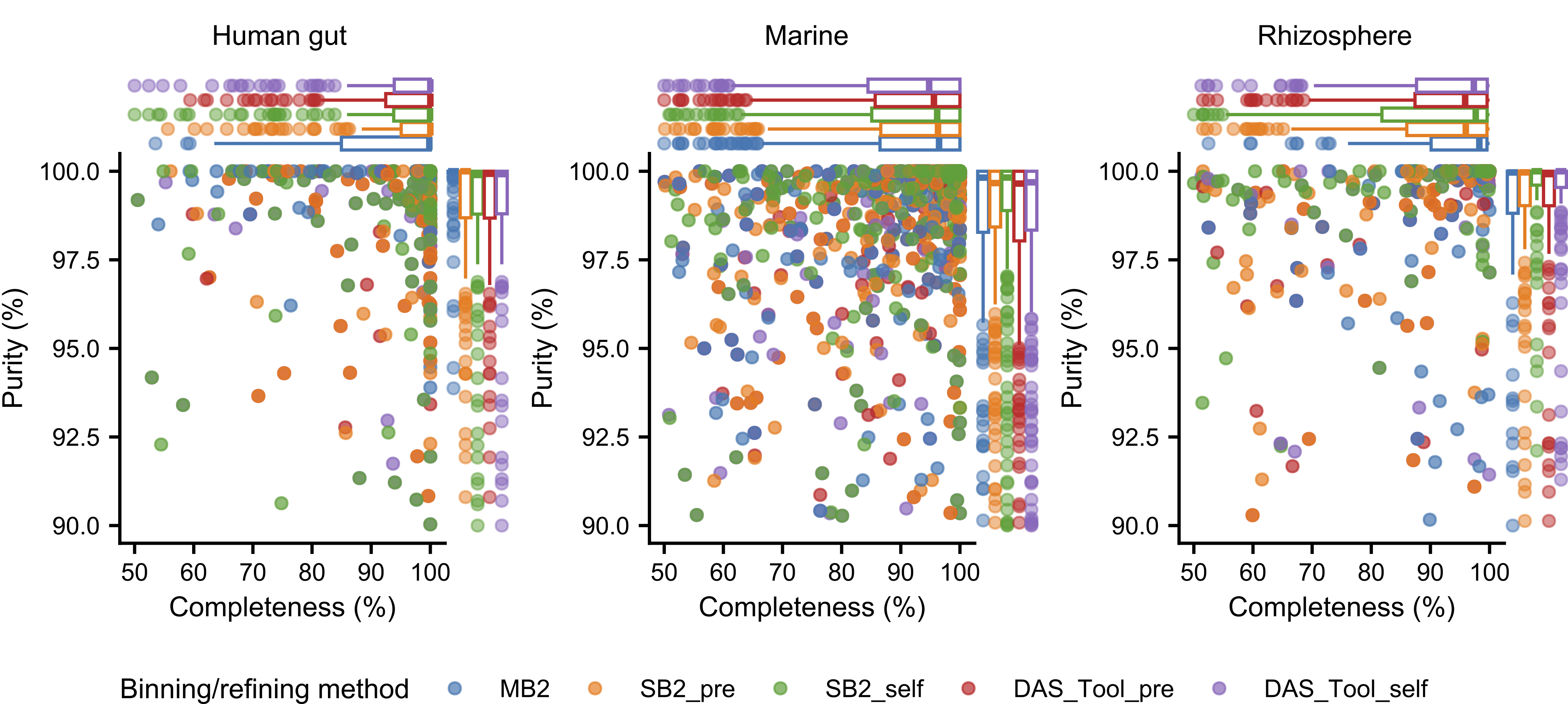
**

**Supplementary Figure 2. Genome size distribution and quality assessment of MAGs across environments and binning/refining methods.** Five different binning/refining combinations were evaluated: MetaBat2 (MB2, blue), SemiBin2 using a prebuilt model (SB2_pre, orange), SemiBin2 using self-supervised learning (SB2_self, green), DAS Tool combining MB2 and SB2_pre (DAS_Tool_pre, red), and DAS Tool combining MB2 and SB2_self (DAS_Tool_self, purple). All MAGs meet minimum quality thresholds of completeness ≥50% and purity ≥90%. Relationship between completeness and purity scores for individual MAGs, with scatter plots showing the distribution of MAGs across different quality thresholds. Box plots on the margins show the overall distribution of completeness (vertical) and purity (horizontal) values for each binning/refining method.


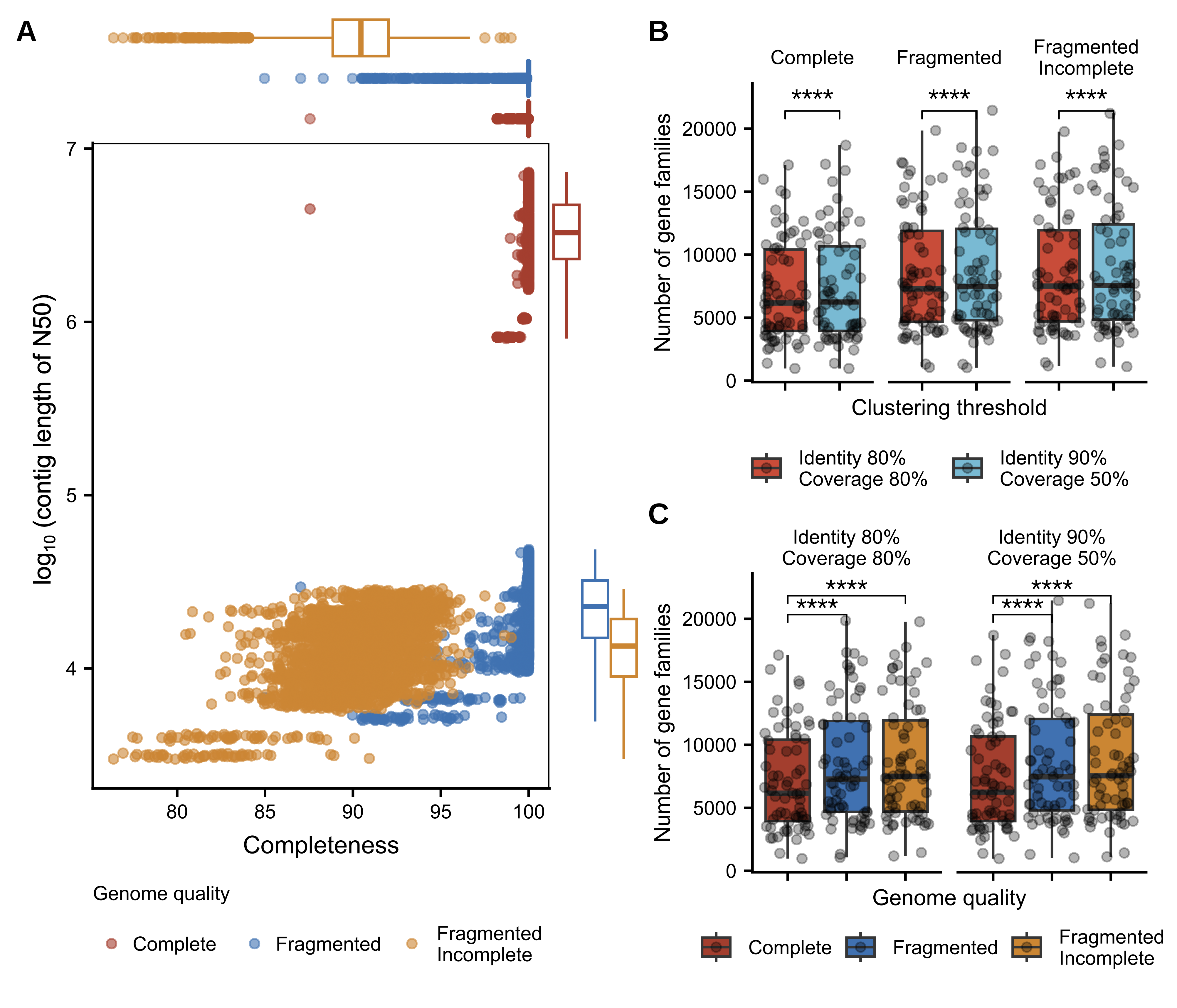


**Supplementary Figure 3. Quality of simulated genome datasets and the number of gene families in pangenomes.** One original complete genome dataset and two MAG-simulated datasets were generated from complete-level NCBI assembly statistics. Simulated datasets were compared to the complete genome dataset. **A** Completeness scores of CheckM2 and log N50 values of genomes. The colors indicate the quality of the genome dataset. The number of gene families of 67-species pangenomes dissected by gene family clustering threshold and genome dataset quality. **B** The effect of gene clustering threshold on the number of gene families in each species across three genome datasets. **C** The effect of genome quality on the number of gene families across gene family threshold clustering. Each point represents the number of gene families in a single species pangenome. Statistical tests were performed and represented above box plots (*P* < 0.0001: ****; paired *t*-test).

**
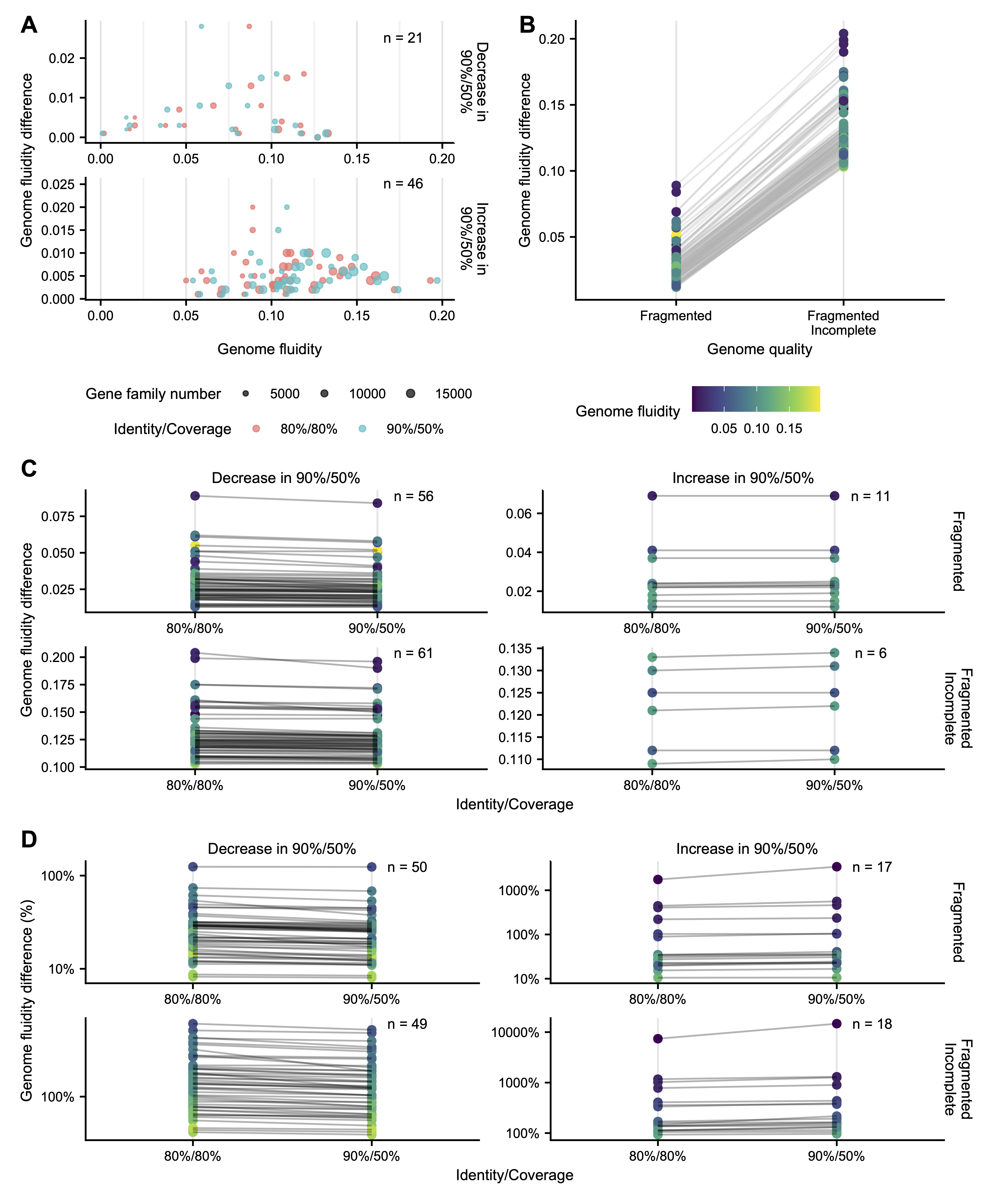
**

**Supplementary Figure 4. Performance evaluation of the gene clustering thresholds for genome fluidity inference.** All points are the original and relative genome fluidity values of species. **A** The effect of gene family clustering threshold on genome fluidity. 67 species of a complete genome dataset were used. Each point size represents the gene family number of a species and color represents the gene clustering threshold. The genome fluidity difference was calculated within a species with varying gene clustering thresholds. The number of species representing genome fluidity difference pattern is shown in each panel. **B** The genome fluidity of "Fragmented" and "Fragmented Incomplete" datasets. Each point color represents the genome fluidity value (y-axis) and the same species are connected by a line. **C** and **D** The effect of the gene clustering threshold on genome fluidity using (**C**) absolute and (**D**) relative difference. The absolute difference is based on the raw genome fluidity value with the respective genome quality dataset. The genome fluidity difference (%) represents the absolute value divided by the original value of the complete genome dataset in the respective gene family clustering threshold in a species, multiplied by 100. The same species are connected by a line.

**
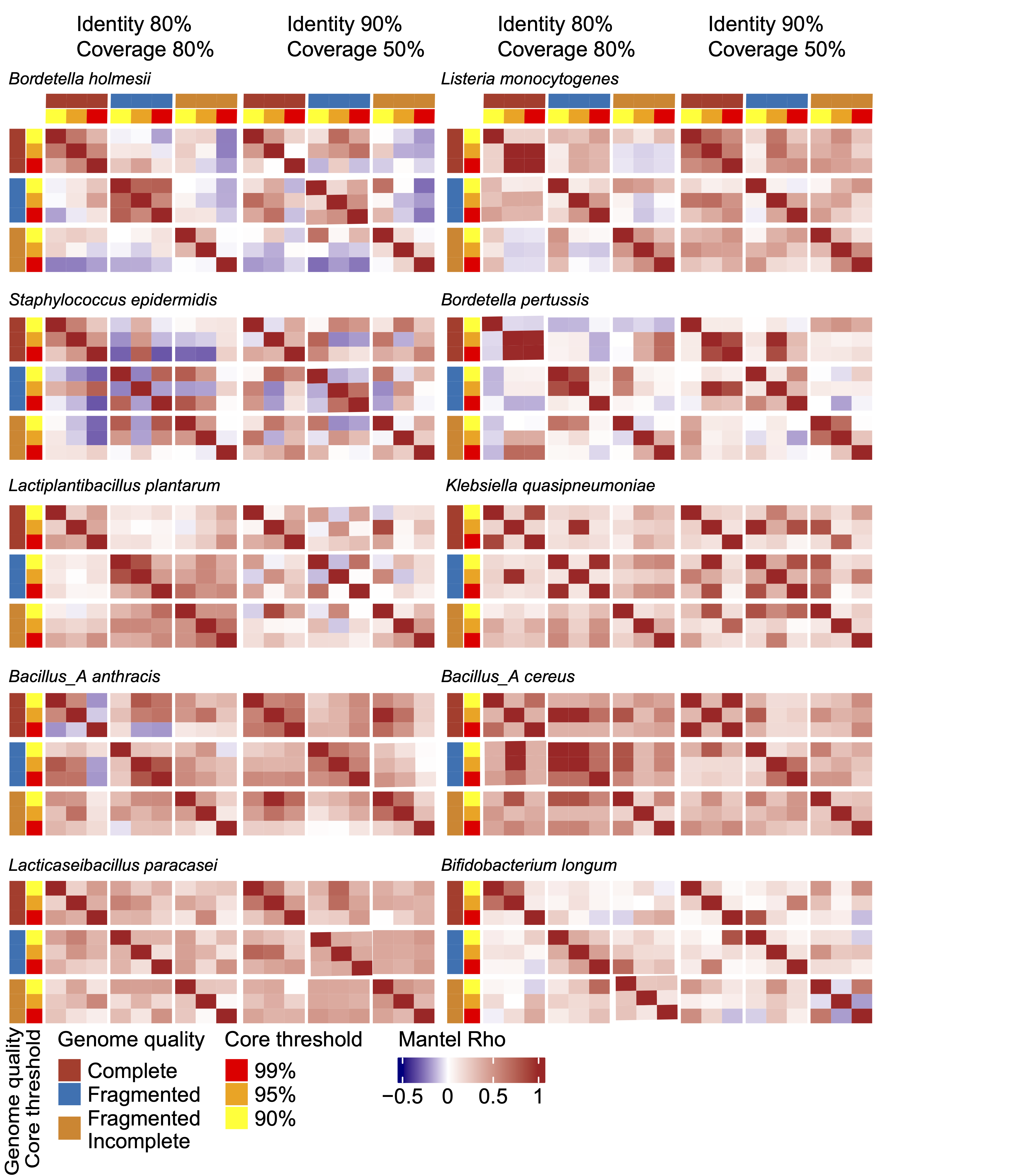
**

**Supplementary Figure 5. Performance evaluation of the gene family clustering thresholds, genome qualities, and core gene thresholds for phylogenetic tree inference.** Similarity matrix of the core genome phylogenetic trees among three genome quality datasets. Core phylogenetic trees were generated using three genome quality datasets from ten species. Pairwise Mantel test was performed based on the patristic distance matrices between each tree pair. Two lines of cell represented on the horizontal and vertical boundary of matrices are the quality of genome datasets ("Complete" - reddish brown, "Fragmented" - blue, "Fragmented Incomplete" - brownish orange) and core gene threshold ("99%" - red, "95%" - orange, "90%" - yellow). For each species, Mantel Rho matrices are shown as 9 × 9 symmetric matrices for each gene family clustering threshold.

**
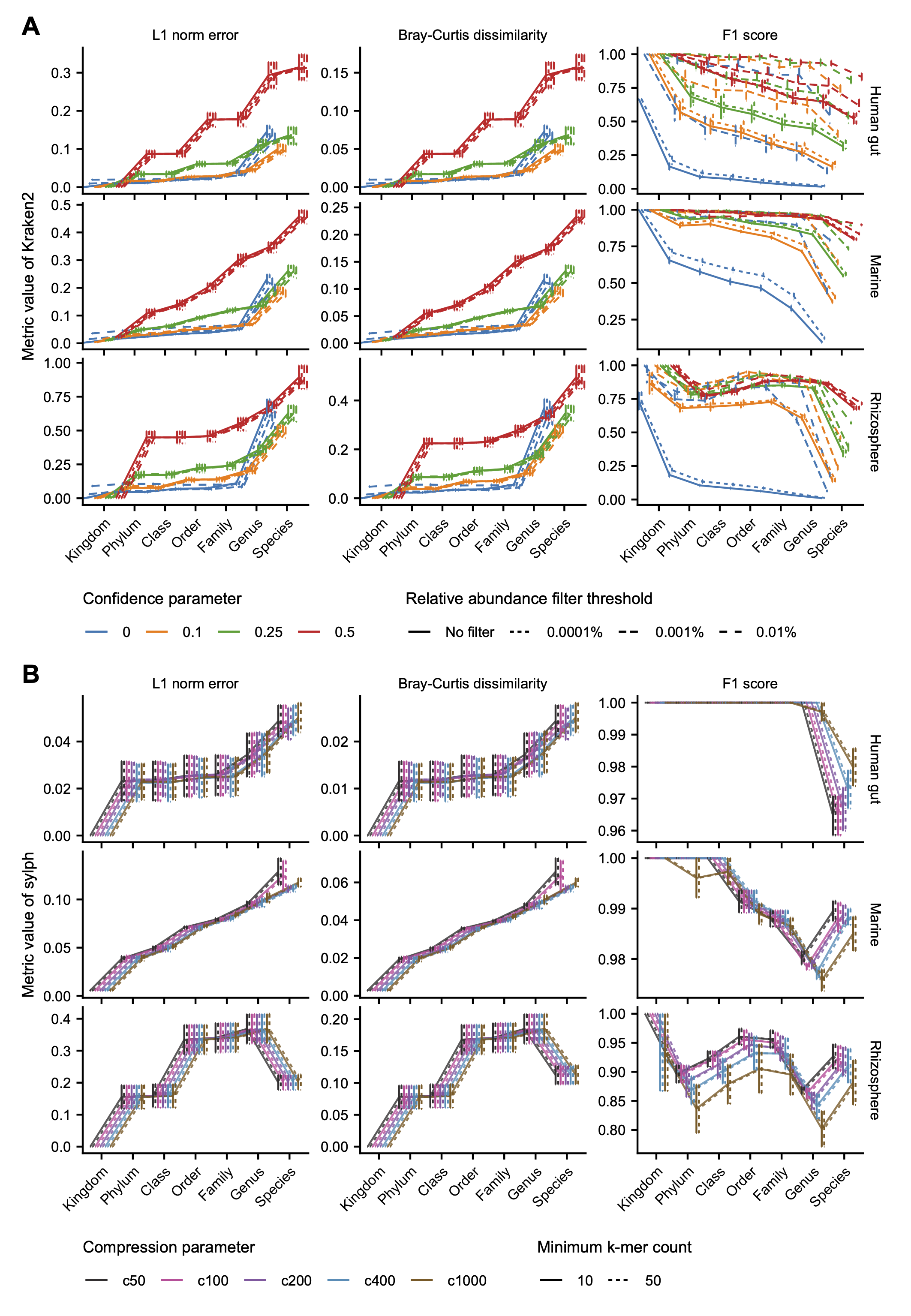
Supplementary Figure 6. Performance evaluation of the parameters of Kraken, Bracken, and sylph for taxonomic profiling accuracy.** Sequence abundance profiling accuracy was evaluated using three distinct environments with three metrics calculated by OPAL. **A** The effects of varying confidence parameters and relative abundance filtering thresholds on profiling accuracy using taxonomic profiles generated by Kraken2 with Bracken. **B** Sylph performance assessment using varying values of compression parameter and minimum number of *k*-mers. All evaluations were performed across taxonomic ranks from Kingdom to Species levels, with metrics including L1 norm error, Bray-Curtis dissimilarity, and F1 score. L1 norm error and Bray-Curtis dissimilarity represent community distance to the gold standard profile and F1 score represents the accuracy of profiled taxa.

**
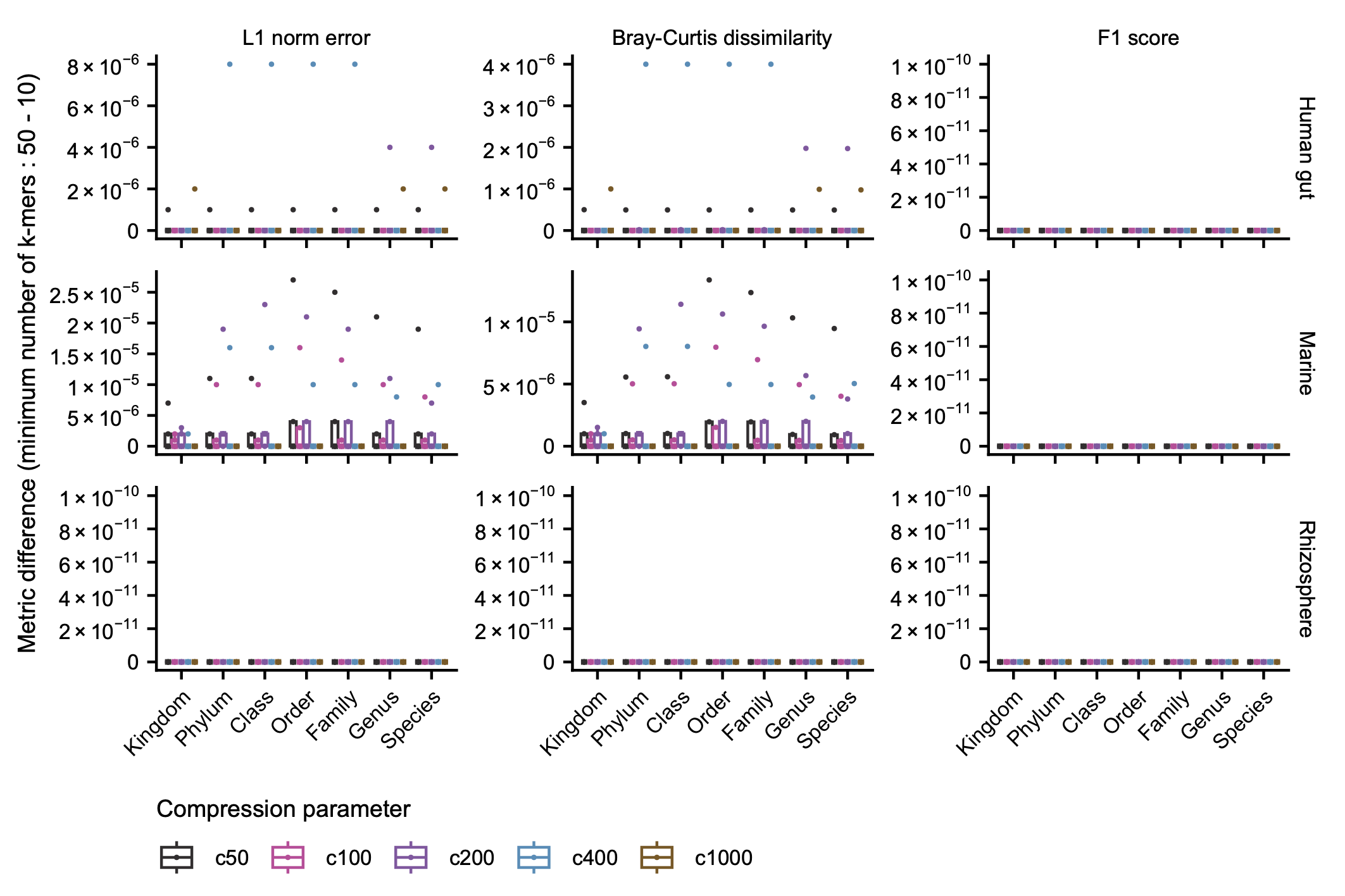
**

**Supplementary Figure 7. Performance evaluation of the minimum number of *k*-mers parameter of sylph for taxonomic profiling accuracy.** The metric difference was calculated between the minimum number of *k*-mer of 10 and 50 across three environments. The minimum number of *k*-mers defines the species existence threshold. The x-axis represents taxonomic ranks from Kingdom to Species, and the y-axis shows the difference in each metric value. Each box plot indicates the distribution of five samples for each condition.

**
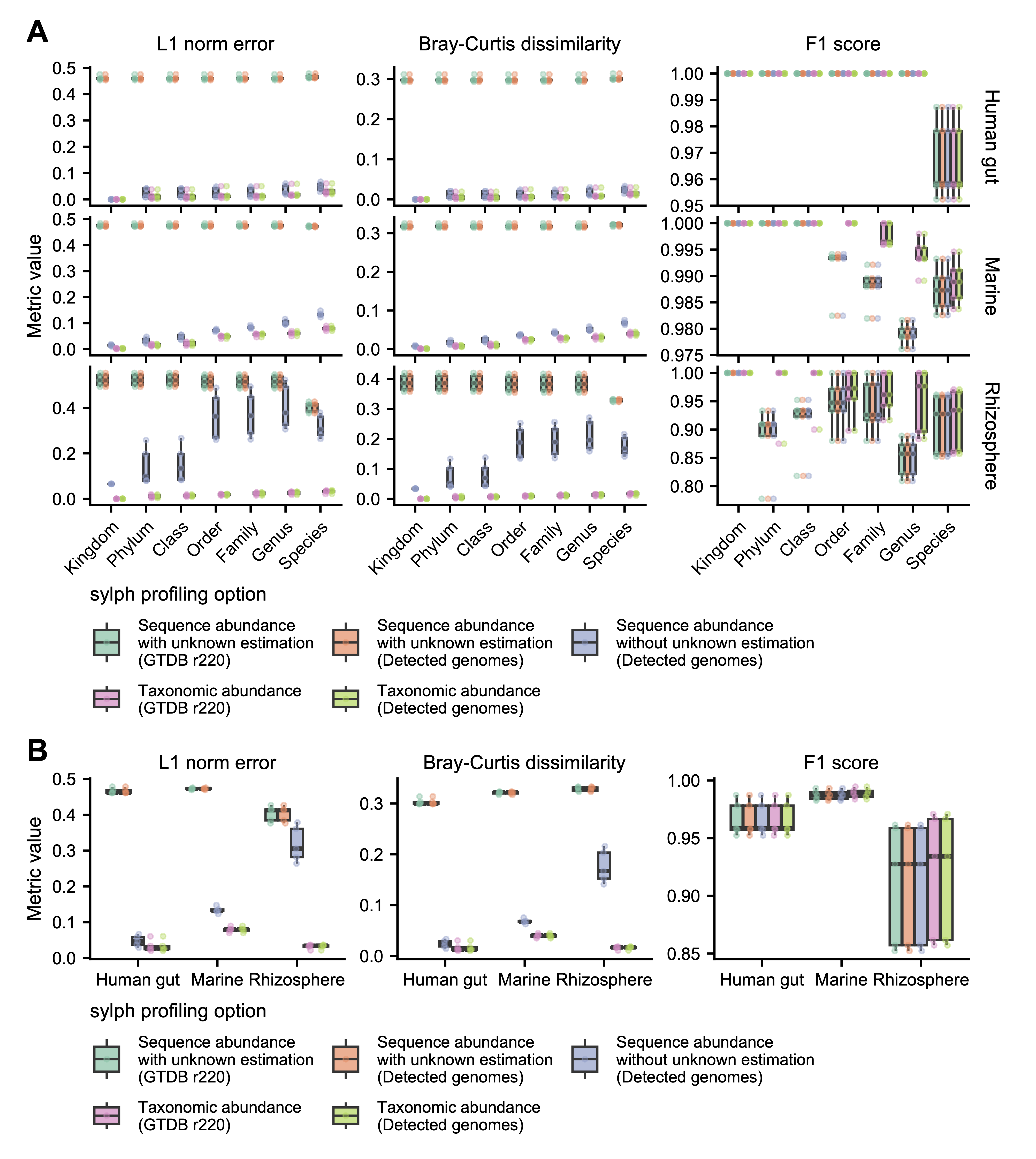
**

**Supplementary Figure 8.** **Performance evaluation of the database coverage and unknown estimation parameter of sylph for taxonomic profiling accuracy.** Sequence abundance and taxonomy abundance profiles were compared to the matching type of gold taxonomic profile to calculate metric values. **A** and **B** The effect of varying sylph parameters, database coverage, and abundance profile type on taxonomic profiling accuracy. The profiling accuracy was assessed based on entire taxonomic ranks (**A**) and species rank (**B**) with L1 norm error, Bray-Curtis dissimilarity, and F1 score.


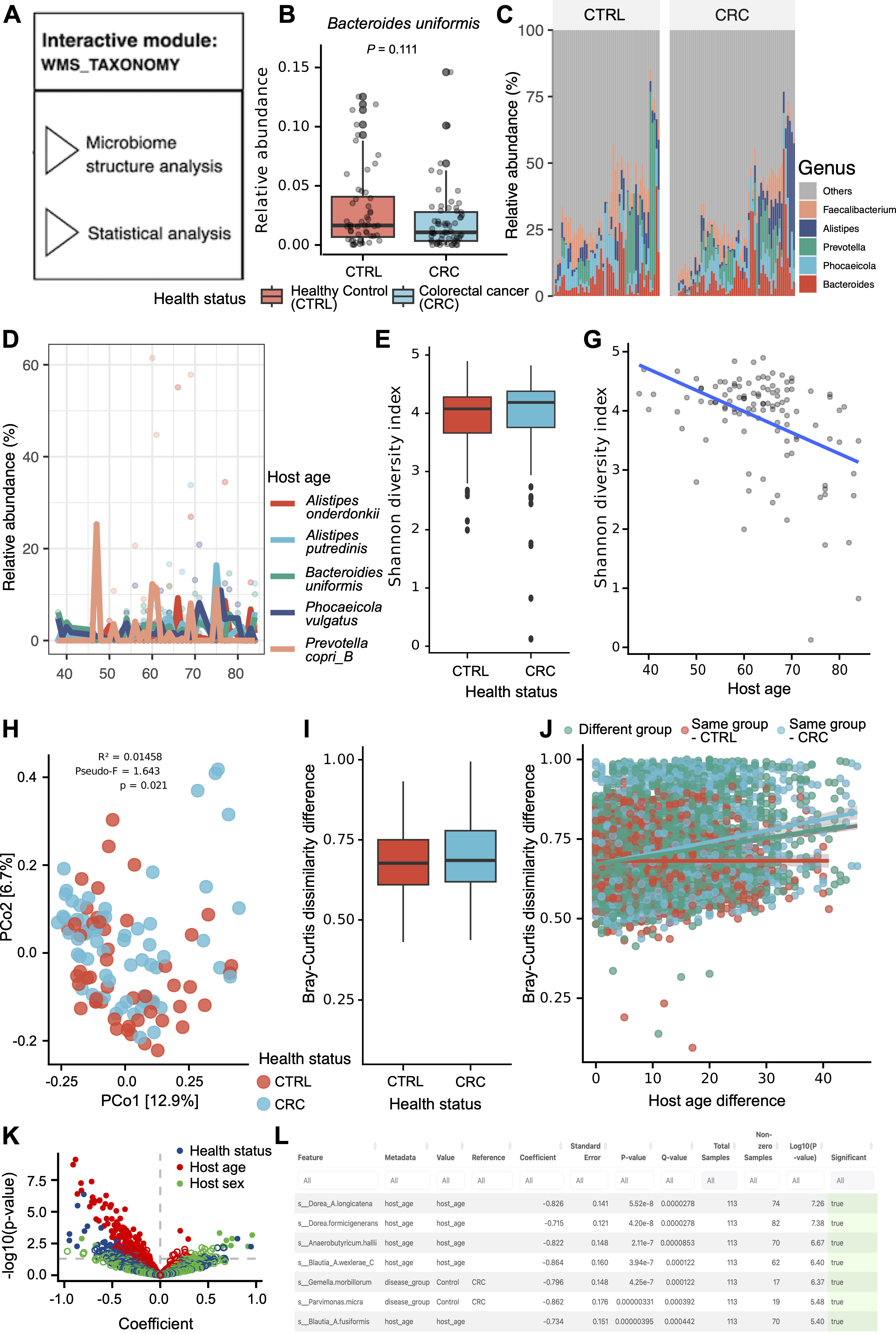


**Figure S9. Functionality of the interactive module that utilizes WMS_TAXONOMY.** **A** The interactive module based on WMS_TAXONOMY. This module executes multiple analyses using the phyloseq object generated in WMS_TAXONOMY. Relative abundance, log transformed, and centered log-ratio transformed abundance can be visualized with selected metadata. All features could be analyzed and visualized from kingdom to species levels. Results of 113 metagenomic data analysis using this module were visualized. **B** Single taxon abundance visualization in box plot. **C** Taxonomic abundance bar plot at the genus level. **D** Relative abundance line plot based on numerical metadata. **E** and **F** Alpha diversity index visualization using the Shannon diversity index with (**E**) categorical and (**F**) numerical metadata variable. **G** PCoA plot of beta diversity using the Bray-Curtis dissimilarity index with PERMANOVA regarding provided metadata variable. **H** and **I** Intra-group Bray-Curtis dissimilarity index comparison with (**H**) categorical and (**I**) numerical variable. **J** and **K** Results of differential association analysis showing (**J**) a volcano plot with statistical significance and effect size across entire metadata variables and (**K**) a visualization of MaAsLin2 association results.


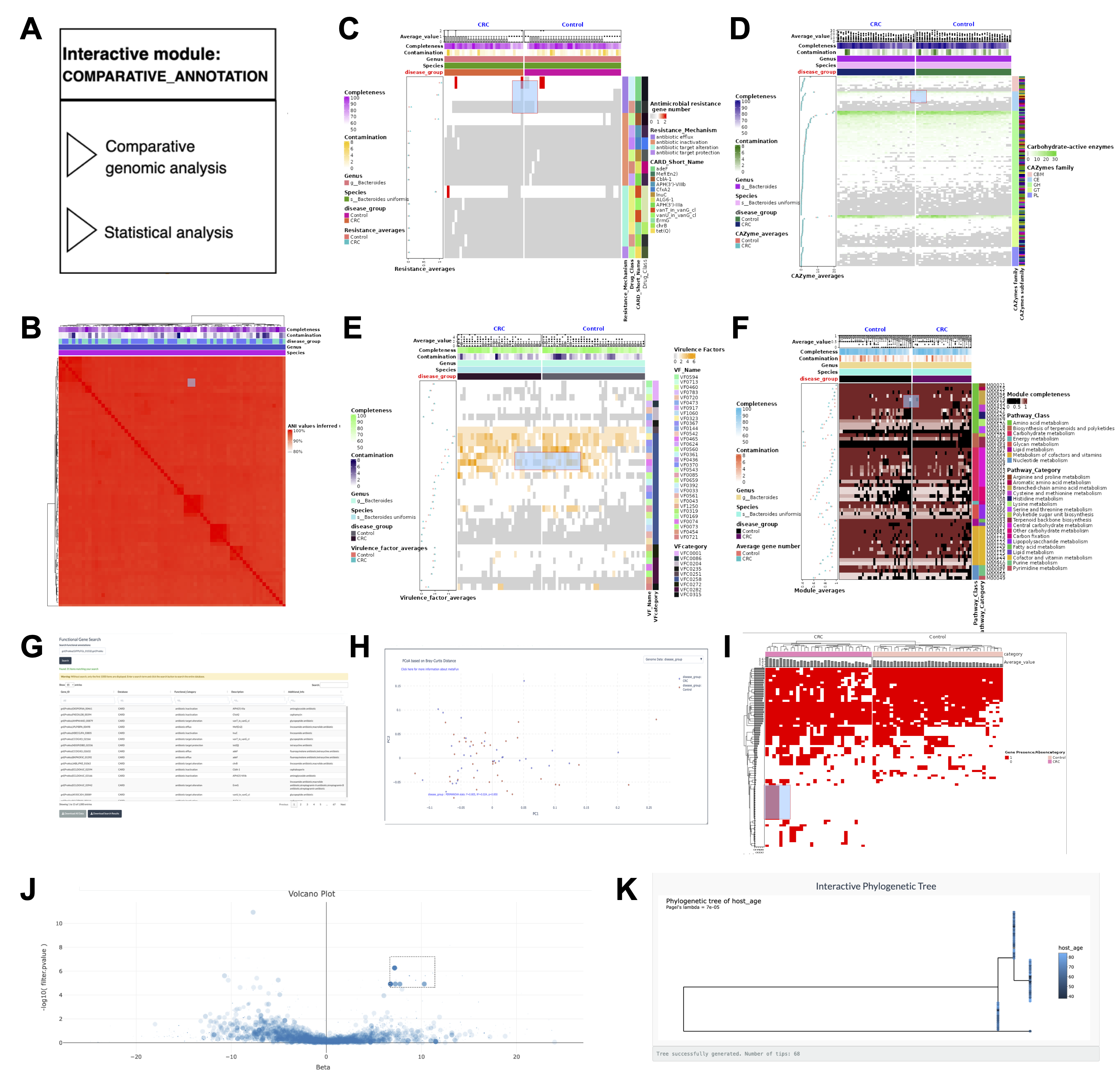


**Figure S10. Functionality of the interactive module that utilizes COMPARATIVE_ANNOTATION.** **A** The interactive module based on COMPARATIVE_ANNOTATION. This module executes multiple analyses using annotation results from COMPARATIVE_ANNOTATION featuring built-in interactive analyses. Results of interactive module analysis using 69 *Bacteroides uniformis* strains were visualized. **B**-**F** (**B**) Interactive heatmap of pairwise average nucleotide identity. Interactive heatmaps for (**C**) antimicrobial resistance genes, (**D**) carbohydrate enzymes, (**E**) virulence factors, and (**F**) metabolic genes with KEGG Orthology annotation. **G** Functional search interface that can identify all annotated functions using COMPARATIVE_ANNOTATION. **H** PCoA plot using gene family presence information. **I** and **J** Interactive visualization of association analysis results using (**I**) categorical variable and (**J**) numerical variable. **K** Interactive visualization of a phylogenetic tree inferred within this module.

**
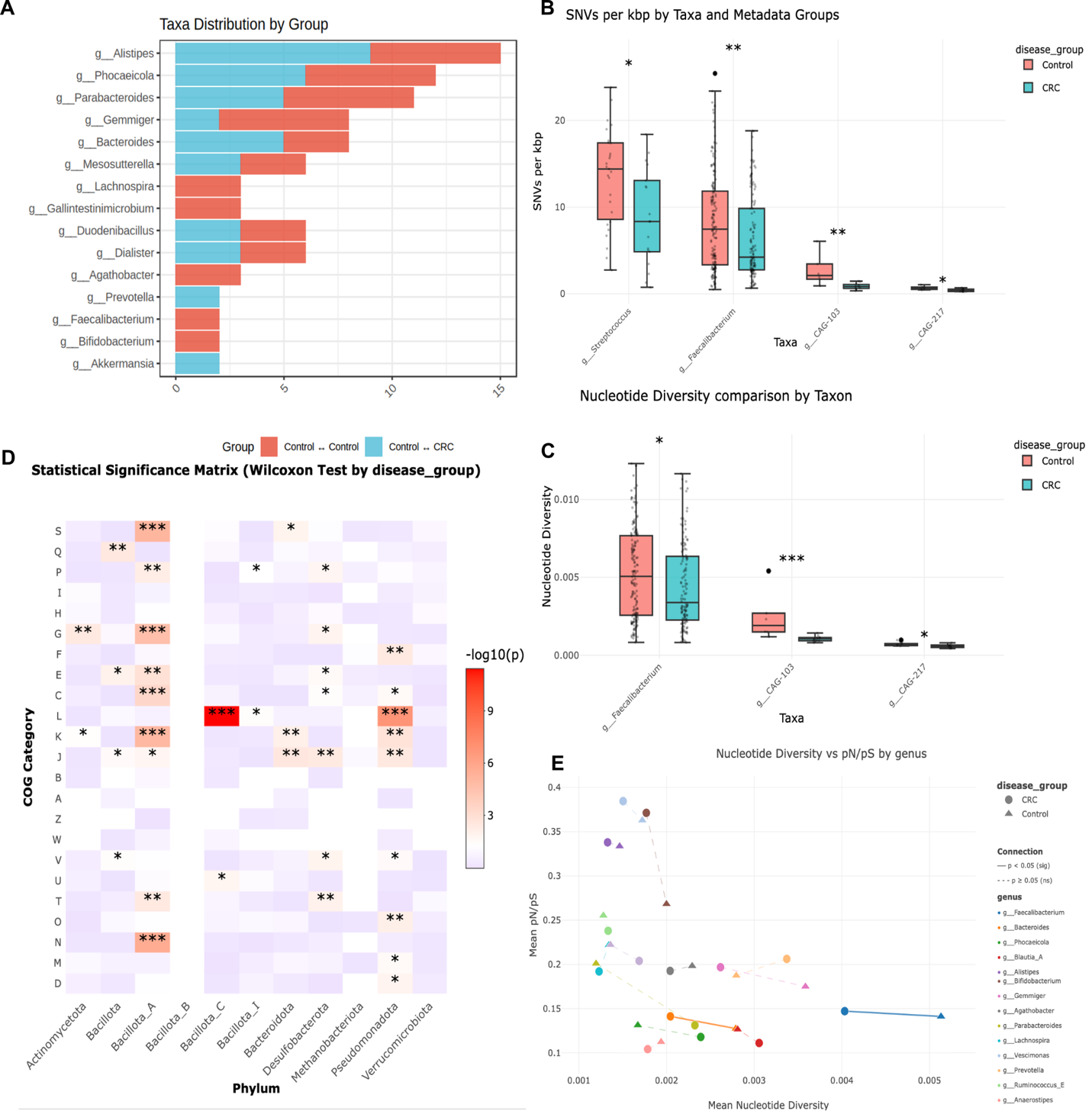
Figure S11. Case analysis of the interactive module INTERACTIVE_STRAIN.**

**A** Taxa distribution showing the number of shared genomes above population ANI 99.999% across Control and CRC samples. **B** SNV density (SNVs per kbp) difference across taxa stratified by disease group with statistical significance assessed using Wilcoxon rank-sum test (* *P* < 0.05, ** *P* < 0.01). **C** Nucleotide diversity (π) of genera with significant differences between disease groups using Wilcoxon rank-sum test (* *P* < 0.05, *** *P* < 0.001). **D** Matrix of pN/pS ratio differences between Control and CRC across COG functional categories and phyla based on gene level. Color intensity represents -log10(P) values with Wilcoxon rank-sum test (* *P* < 0.05, ** *P* < 0.01, *** *P* < 0.001). **E** Scatter plot of mean nucleotide diversity versus mean pN/pS ratio by genus based on genome level. Solid lines connect paired samples with significant differences (*P* < 0.05; Wilcoxon rank-sum test); dashed lines indicate non-significant comparisons.


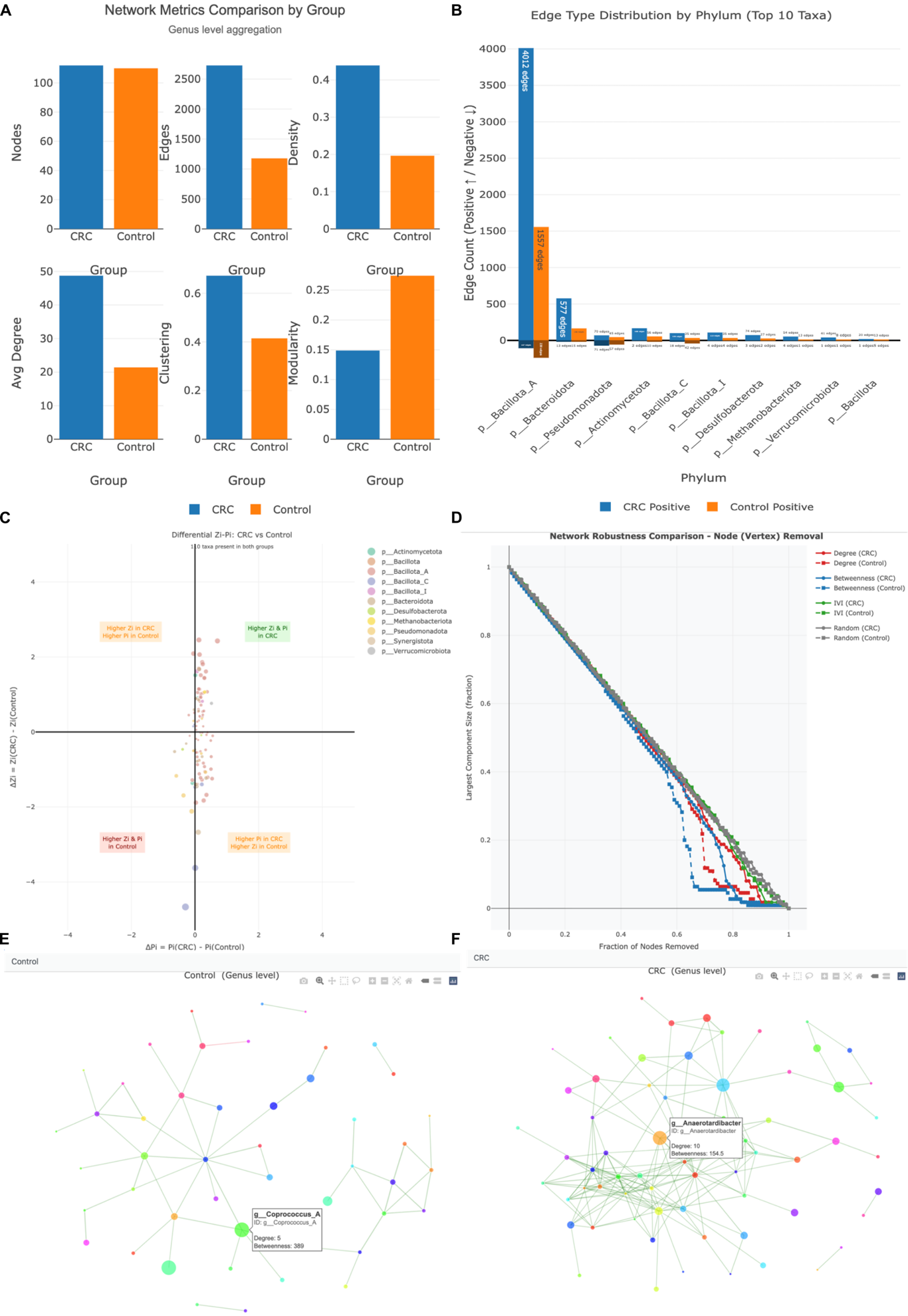


**Figure S12. Case analysis of the interactive module INTERACTIVE_NETWORK.**

**A** Network topology metrics comparison between CRC and Control groups at genus level, including node count, edge count, density, average degree, clustering coefficient, and modularity. **B** Edge type distribution by phylum showing positive and negative correlation counts for top 10 taxa. **C** Differential Zi-Pi plot comparing within-module connectivity (ΔZi) and participation coefficient (ΔPi) between CRC and Control networks. **D** Network robustness comparison under targeted (degree, betweenness, IVI) and random node removal strategies, showing largest connected component size as a function of fraction of nodes removed. **E** and **F** Interactive network visualization at genus level for (**E**) Control and (**F**) CRC groups. Node size represents Integrated Value of Influence and node color indicates genus.
